# Dynamic Changes in Microvascular Flow Conductivity and Perfusion After Myocardial Infarction Shown by Image‐Based Modeling

**DOI:** 10.1161/JAHA.118.011058

**Published:** 2019-03-22

**Authors:** Polyxeni Gkontra, Wahbi K. El‐Bouri, Kerri‐Ann Norton, Andrés Santos, Aleksander S. Popel, Stephen J. Payne, Alicia G. Arroyo

**Affiliations:** ^1^ Centro Nacional de Investigaciones Cardiovasculares (CNIC) Madrid Spain; ^2^ Biomedical Image Technologies (BIT), ETSI Telecomunicación, Universidad Politécnica de Madrid Madrid Spain; ^3^ Institute of Biomedical Engineering Department of Engineering Science University of Oxford United Kingdom; ^4^ Division of Science, Mathematics, and Computing Bard College Annandale‐on‐Hudson NY; ^5^ Centro de Investigación Biomédica en Red de Bioingeniería Biomateriales y Nanomedicina (CIBERBBN) Madrid Spain; ^6^ Department of Biomedical Engineering School of Medicine Johns Hopkins University Baltimore MD; ^7^ Centro de Investigaciones Biológicas (CIB‐CSIC) Madrid Spain

**Keywords:** blood flow, confocal microscopy, coronary microcirculation, mathematical modeling, myocardial infarction, Computational Biology, Hemodynamics, Ischemia, Myocardial Biology

## Abstract

**Background:**

Microcirculation is a decisive factor in tissue reperfusion inadequacy following myocardial infarction (MI). Nonetheless, experimental assessment of blood flow in microcirculation remains a bottleneck. We sought to model blood flow properties in coronary microcirculation at different time points after MI and to compare them with healthy conditions to obtain insights into alterations in cardiac tissue perfusion.

**Methods and Results:**

We developed an image‐based modeling framework that permitted feeding a continuum flow model with anatomical data previously obtained from the pig coronary microvasculature to calculate physiologically meaningful permeability tensors. The tensors encompassed the microvascular conductivity and were also used to estimate the arteriole–venule drop in pressure and myocardial blood flow. Our results indicate that the tensors increased in a bimodal pattern at infarcted areas on days 1 and 7 after MI while a nonphysiological decrease in arteriole–venule drop in pressure was observed; contrary, the tensors and the arteriole–venule drop in pressure on day 3 after MI, and in remote areas, were closer to values for healthy tissue. Myocardial blood flow calculated using the condition‐dependent arteriole–venule drop in pressure decreased in infarcted areas. Last, we simulated specific modes of vascular remodeling, such as vasodilation, vasoconstriction, or pruning, and quantified their distinct impact on microvascular conductivity.

**Conclusions:**

Our study unravels time‐ and region‐dependent alterations of tissue perfusion related to the structural changes occurring in the coronary microvasculature due to MI. It also paves the way for conducting simulations in new therapeutic interventions in MI and for image‐based microvascular modeling by applying continuum flow models in other biomedical scenarios.

**Clinical Perspective**

**What Is New?**

- This study is the first to quantify perfusion‐related changes at different stages after myocardial infarction using 3‐dimensional anatomical data of the porcine cardiac capillary bed and image‐based modeling.
- Changes in microvascular conductivity, drop in pressure, and myocardial blood flow follow a bimodal pattern in infarcted areas with a normalization effect around day 3 after myocardial infarction.

**What Are the Clinical Implications?**

- This study permits going beyond experimental measurements and, thus, enables insights into the function of microcirculation and its alterations after myocardial infarction, as well as the relation with organ‐level flow measurements performed in the clinic.
- Simulations of vascular remodeling will allow identification and experimentation with new therapeutic strategies that could improve microcirculation after myocardial infarction.

## Introduction

The primary function of microcirculation is the supply of oxygen and nutrients by blood flow to the tissues in order to meet their metabolic demands.^1^, ^2^ Dysfunction of microcirculation after myocardial infarction (MI), even after successful reperfusion of coronary arteries, is increasingly being recognized as the determinant of inadequate myocardial reperfusion of tissue, leading to prolonged ischemia and adverse clinical outcomes.^3^ Consequently, assessment of blood flow at the microvascular level is of paramount importance in elucidating the relation between the structure of the microcirculation and effective fluid and mass transport for improvement of therapies. Although advances in imaging systems, in combination with computational image analysis approaches, have facilitated reconstruction of detailed 3‐dimensional (3D) microvascular networks,^4^, ^5^, ^6^, ^7^ in vivo measurement of blood flow or pressure in microvessels embedded in the deeper layers of organs, such as the heart, is a particularly challenging task and feasible for only some epicardial vessels.^8^ In an effort to go beyond experimental observations, mathematical models of blood flow in microvascular networks have complemented experimental approaches. These models have the potential of offering unprecedented insight into perfusion and structure–function relations by exploiting structural data.^9^, ^10^

Microvascular blood flow models can be roughly categorized into discrete and continuum flow models (CFMs). Models within the first category provide important information regarding distribution of hemodynamics, and they have applications in a variety of tissues, including the rat mesentery,^11^ brain,^12^ tumor,^13^ and heart.^14^ Nonetheless, they require prescription of adequate boundary conditions (BCs), namely, flow or pressure, at all boundary nodes of the network. Several work‐arounds have been adapted such as assigning constant or scaled pressure values according to the vessel diameter or type,^14^, ^15^, ^16^ imposing no‐flow BCs,^17^ and, more recently, using optimization methods.^18^, ^19^ However, the effect of BCs on the estimated blood flow cannot be underestimated. Lorthois et al^12^ studied this effect and demonstrated the strong dependence of the final flow solution on the chosen BCs for boundary capillaries using the brain microvascular data reconstructed by Cassot et al.^4^

The limitation of prescribing adequate BCs to the thousands of nodes usually present in microvascular networks can be tackled using CFMs. CFMs do not allow simulation of blood flow at individual microvessels. They do, however, permit prediction of tissue‐scale blood flow properties and thus have started to gain attention as an alternative to discrete models.^20^ This type of model is based on Darcy's law, which describes fluid flow in porous media and defines *flow* as the product of pressure gradient with the permeability of the media.^21^ In this context, *permeability* refers to the inverse resistivity of flow (ie, network conductivity), and in 3D structures, it is expressed by a tensor. The permeability tensor is a tissue‐scale metric that relates the microscale topology, that is, the topology of the microvascular bed, to the macroscale flow. Thus, it provides a way to quantify the effect of structural changes that occur as a result of microvascular diseases and to relate these to whole‐organ flow observations.^22^, ^23^ This aspect, coupled with the computational efficiency of CFMs and their independence of BCs, makes such models a powerful tool in advancing our understanding of tissue perfusion in healthy and pathological conditions and in elucidating structure–function relations. Moreover, they permit scaling up of discrete models, using permeability tensors to parameterize multiscale models of fluid transport in complete organs, such as the heart.^9^

Permeability tensors can be efficiently calculated using homogenization theory. In particular, Shipley et al^24^ adapted homogenization to develop a continuous model of fluid and drug transport for a tumorous, spatially periodic, leaky capillary bed. The model was later adapted by Smith et al^22^ to calculate physiologically relevant permeability tensors for the study of transmural variations in a nonleaky rat myocardium capillary bed, generated statistically from anatomical data.^6^ A similar approach was followed by El‐Bouri and Payne^25^ to calculate permeability tensors for different sizes of 3D, statistically generated, spatially periodic, brain microvascular networks, leading to a converged permeability tensor that can be used to scale up larger areas of the brain microvasculature to understand whole‐organ vascular flow. Hyde et al^16^, ^26^ recently compared different approaches to parameterize a continuous multicompartment model that permits calculation of a flow solution at each compartment and integration of the models toward building a multiscale continuum perfusion model of the whole heart, with applications in porcine and canine animal models. The different parameterization methods were based on approximations of the permeability tensor using various degrees of anatomical information. These works represent the first efforts to use anatomical data instead of idealized structures in statistically or rule‐based generated networks. In these works, however, the tensors were not calculated by solving Darcy's law directly on the vascular networks, as this task is rather challenging when using anatomical data.

In this study, we adapted a CFM approach to assess the effect of MI and its subsequent progression (1, 3, and 7 days after MI) on microvascular perfusion. In an earlier study of MI at the same time points,^7^ we discovered that the microvasculature in infarcted areas progressively lost its integrity and complexity. The structural remodeling of microvessels 7 days after MI, expressed by the presence of larger vessels and loss of capillaries, among other details, became intense and led to worsening efficiency in oxygen supply, as indicated by larger intercapillary (extravascular) distances. In contrast, infarcted areas 3 days after MI were closer to the basal compared with 1 or 7 days after MI, indicating normalization of the effect of infarction around that day. Changes in noninfarcted (remote) areas were absent or milder than those observed in the infarcted areas, but as pathology progressed, they appeared to intensify. Our aim in this work was to unravel the functional implications of these microvascular structural changes that occur in response to MI by calculating permeability tensors.

To investigate these functional implications, we used the anatomical data from thick slices of cardiac tissue extracted from the pig animal model used in our previous study. We developed an image‐based modeling framework that permits the use of a CFM directly on anatomical data instead of statistically or rule‐based generated networks to calculate physiologically meaningful permeability tensors. We subsequently used the tensors and values from magnetic resonance imaging (MRI) data to estimate the expected drop in pressure (DP) along an arteriole–venule (AV) path. Moreover, we calculated the myocardial blood flow (MBF) in 2 scenarios, assuming (1) the same DP for all tissue conditions and (2) DP values defined according to the condition of the tissue. In the first case, the DP was considered constant. In the second case, we used the mean value of the previously calculated DP per tissue condition. The simulation results indicate the existence of dynamic changes in the permeability tensors, DP, and MBF related to the structural remodeling and altered angioarchitecture of the microvasculature after MI. We also investigated whether these changes persisted 45 days following MI, as was the case with the structural changes that not only persisted but also intensified at this time point.^7^ In addition, the phase separation effect was incorporated into the calculation of the permeability tensors, and its effect was quantified. Last, we quantified the impact that specific modes of vascular remodeling, such as vascular dilatation, constriction, or pruning, had on the permeability tensors.

## Methods

### Data Acquisition and Image Processing

The acquisition and processing of the 3D image data used in this work have been described in detail elsewhere.^7^ In brief, we used thick slices of cardiac tissue (≈100 μm) that had been harvested from the left ventricle of pigs in basal conditions and pigs that had been euthanized 1, 3, and 7 days following MI. MI was induced percutaneously by means of an angioplasty balloon with 30‐minute occlusion of the left anterior descending coronary artery, followed by reperfusion. For the analysis of tissues at day 45 after MI, occlusion was performed for 40 minutes. For subjects that had MI, tissues were obtained from infarcted and noninfarcted (remote) areas. These samples were subsequently stained for cell nuclei (Hoechst), endothelial junctions (anti–vascular endothelial cadherin) and smooth muscle cells (anti–α‐SMA [anti–α–smooth muscle actin]) and were imaged by Leica SP5 confocal microscopy. During imaging, *z*‐stack slices of 1024×1024 pixels were acquired every 1 μm. The number of slices acquired, and thus the size of the resulting image along the *z*‐axis, depended on the depth of antibody penetration. The voxel size was 0.3785×0.3785×1.007 μm, with the exception of a few volumes for which it was 0.3142×0.3142×1.007 μm.

The confocal images were segmented using the multiscale multithresholding algorithm.^27^ The filling approach detailed by Gkontra et al^7^ was applied on top of the segmentations of the vascular endothelial cadherin channel to accurately reconstruct the complete microvasculature from endothelial junctions. Last, the graph‐based representation of the microvasculature, along with the radii and lengths of the microvessels, was extracted from every connected subnetwork that made up the network.

It should be noted that a subset of the complete previous data set was used. The subset contains images of tissue samples that belong solely to the myocardium, excluding images of samples from the endocardium and the epicardium because the orientation of vessels^28^ in those areas would render simulations meaningless. The categorization of the volumes into the 3 regions of the heart was performed by 2 expert biologists after acquisition and based on the orientation of the vessels in the images. Consequently, the subset used in this work consists of 12 images (3D) in basal conditions; 13 images of infarcted conditions on days 1 and 3, respectively, after MI; 15 images of infarcted conditions 7 days after MI; 16 images of remote conditions on day 1 after MI; 11 images of remote areas 3 days after MI; and 9 images of remote areas 7 days after MI. The data set was enriched with data from infarcted and remote areas 45 days after MI that had been acquired and analyzed in a manner similar to the rest of the time points. In all conditions, image volumes came from 2 independent pigs (basal and days 1, 3, and 7 after MI) or 3 independent pigs (45 days after MI; Table S1). For simplicity, in tables in the current work, images corresponding to tissue from infarcted areas at 1, 3, 7, and 45 days after MI were abbreviated as I1MI, I3MI, I7MI, and I45MI, respectively, and images from remote areas were abbreviated as R1MI, R3MI, R7MI, and R45MI, respectively.

### Calculation of Permeability Tensors and Perfusion in Microvascular Networks

Calculation of permeability tensors was performed by applying a continuous 2‐phase porous perfusion model adapted from El‐Bouri and Payne^25^ (Data S1). In brief, we assume a completely connected, spatially periodic network with no blind ends, that is, branches that are connected to only 1 branch and do not touch volume borders. In 3D, the tensor is of size (3×3) and the elements of each row of the permeability tensor can be then calculated by applying a pressure gradient along 1 of the 3 possible directions (ie, *x*‐direction, *y*‐direction or *z*‐direction in Cartesian coordinates) and by calculating the sums of flow rates on the surfaces of the image volume in each case. The diagonal elements account for the link between the flow rate in 1 direction and the pressure gradient in the same direction, whereas the off‐diagonal elements capture the link between the flow rate in other directions (cross‐flow) and the pressure gradient in the 1 direction. More precisely, each element of the permeability tensor with position (*i, j*) for an image volume *I* is calculated as follows:

(1) $$k_{\mathrm{ij}}\left(I\right)=\frac{\sum_{m=1}^{M_{j}}{\text{q}}_{m}^{j}}{{\text{S}}_{j}\nabla {\text{p}}^{i}},\text{i},\text{j}=1,2,3,$$

where ∇*p* ^*i*^ stands for the pressure gradient applied at direction *i*,* M*_*j*_ is the number of vessels on the outflow surface at the direction *j*, and *S*_*j*_ is the surface area of the outflow surface at direction *j*. The abbreviation ${\text{q}}_{m}^{j}$ stands for the blood flow rate of vessel *m*=1, …, *M*_*j*_ at direction *j*. By assuming axial flow, the flux at each microvessel can be calculated according to the Poiseuille law:

(2) $${\text{q}}_{m}^{\;j}=\frac{\pi {\text{r}}_{m}^{4}\Delta {\text{P}}_{m}^{j}}{8\mu_{m}{\text{l}}_{m}},$$

where *r*_*m*_, *l*_*m*_, μ_*m*_, and Δ${\text{P}}_{m}^{j}$ represent the length, radius, viscosity, and DP, respectively, of microvessel *m* on surface *j*. When constant hematocrit is assumed for all vascular segments, viscosity is calculated according to equations of Pries et al.^11^, ^29^ Given the viscosity of the microvessels, the only unknown for calculating the flow ${\text{q}}_{m}^{j}$ of the microvessels—and, therefore, the permeability tensor—is the DP. The latter can be calculated by solving a system of linear equations. More precisely, by applying the pressure gradient Δ${\text{P}}_{m}^{j}$ on the surfaces of the direction *i*=1, and by assuming that nodes on outflow surfaces of the other directions are connected with their spatial periodic nodes of the corresponding inflow surface and that the only open end nodes are on those surfaces, we have a system of linear equations in which the unknown are pressures at the 2 nodes of the microvessels. This way, we can calculate the pressures at all nodes of the system, and thus Δ${\text{P}}_{m}^{j}$ and ${\text{q}}_{m}^{j}$ for all microvessels, subsequently used to calculate *k*_11_, *k*_12_, *k*_13_. The process is repeated for the other 2 directions.

Last, by assuming that Darcy velocity is parallel to one of the principal directions of flow, and taking into account the relation between perfusion and the permeability tensors and the relation between perfusion and MBF (Data S1),^30^ the latter in a region of the myocardium can be calculated using the permeability tensors:

(3) $$\begin{array}{cc} & \text{MBF}\left(\text{mL/min per 100 g}\right)\\ & =\frac{k_{\mathrm{ii}}\times \Delta p\times 0.133\times 10^{-3}\times 60\times 100}{\rho \times l^{2}}, \end{array}$$

where *k*_*ii*_ represents one of the diagonal elements of the permeability tensor **K**(I) (*i*=1, 2, 3); Δ*p* stands for the AV DP in millimeters of mercury over a microvascular path of length l given in micrometers; ϱ represents myocardial density (ϱ); and 0.133×10^−3^, 60, and 100 are conversion factors for pressure units from millimeters of mercury to kilograms per micrometer per second squared, from minutes to seconds, and from 1 to 100 g, respectively.

### Dealing With Real Anatomical Data: The Infarcted Pig Microvasculature

Completely connected and spatially periodic networks are required in order to perform blood flow simulations. This permits the calculation of flow rate on the surfaces of the tissue by applying a pressure gradient in only 1 direction and assuming that nodes on other surfaces are periodic, as explained earlier. Therefore, when dealing with anatomical data, the first issue that needs to be tackled is the presence of disconnections in microvascular networks due to sample preparation, imaging, and/or reconstruction processes. In recent years, different algorithms have been proposed to correct for missing vascular connectivity.^31^, ^32^, ^33^ Nonetheless, when dealing with the cardiac microvasculature after MI, safe assumptions cannot be made for recovering connectivity because the disconnections that are present might be pathology related and thus should be preserved. For this reason, a different approach has been developed. This approach uses connected components analysis, which is applied to the microvasculature to label the fully connected subnetworks that compose it. Subnetworks that represent at least 15% of the complete vascular density are retained, whereas the rest are not considered in subsequent simulations. For each subnetwork, its skeleton is calculated by means of a thinning procedure.^34^

The next step is to eliminate blind ends from the microvascular networks. This is done based on the assumption that blind ends are possible sprouts that would not contribute to the permeability tensors or are imaging/skeletonization artifacts. It should be noted that before this pruning step, end branches located within a small distance from volume borders are elongated to reach the border and thus avoid being pruned as blind ends. This additional step is incorporated into the process to avoid discrepancies in imaging and skeletonization procedures that lead to branches that do not touch image borders but rather end within a few micrometers of them.

The application of the aforementioned steps for processing the initial volumes results in volumes that might be smaller than the original volumes. In an effort to identify sizes of volumes below which calculation of permeability tensors could lead to errors, we studied the permeability tensors in relation to the size of the resulting subnetworks (Data S1). Such a step is important because application of homogenization techniques is valid only when the length of the microcell unit is large enough to permit calculation of a converged permeability tensor that remains constant within the volume. In fact, the smallest size of spatially periodic units that results in the calculation of the converged/effective permeability tensor is known as the representative elementary volume.^21^ Peyrounette et al^35^ developed a hybrid model and applied it to cubic volumes of brain microvascular data. It was observed that errors increased with a decreasing ratio of side length of volume per capillary length and stabilized at a ratio of ≈4, which corresponds to volumes with side length equal to 200 μm. Smith et al^30^ also observed increased permeability tensor values for microcell units with side length <200 μm, whereas El‐Bouri and Payne^25^ estimated the side length of the representative elementary volume at 375 μm. In this work, we found a cutoff side length for the representative elementary volume along *x–y* of 170 μm. Therefore, volumes below this side length were excluded from subsequent analysis.

Because anatomical data do not present spatial periodicity, as assumed by CFM, we introduce a mirroring solution that permits direct use of CFM on anatomical data. More precisely, the initial volume *I* of size [*Nx, Ny, Nz*], which contains a fully connected microvascular subnetwork, is mirrored along *x*‐direction. The resulting volume is then mirrored along *y*‐direction, and, finally, mirroring of the already‐mirrored volume along the first 2 dimensions takes place along z‐direction. This procedure results in an image volume of size [2×*Nx*, 2×*Ny*, 2×*Nz*] that contains 8 flipped duplicates of *I*. Nodes on the opposite face of *I* along all directions are thus periodic (Figure S1). Before moving to the next step, we validated that our mirroring approach did not affect calculation of the permeability tensors. For this purpose, we performed a second mirroring of the mirrored volumes, and tensors for the 2 cases (ie, mirroring once and twice) were compared. The results indicated that the permeability tensors were almost identical in both cases, confirming the appropriateness of the approach in dealing with the lack of periodicity in anatomical data and in paving the way for blood flow simulations using Darcy CFMs in the case of such data sets.

After mirroring each subnetwork, the permeability tensors of each subnetwork were calculated and subsequently fused to produce the permeability tensors of the volume. Fusing of the tensors of the subworks was inspired by developments for fusing the tensors of heterogeneous media to scale up from the tensors of individual phases of which the media consists. Such approaches include the harmonic average for serial flow, the weighted average for parallel flow, and the geometric average for random flow.^36^ We have adapted the weighted average because the flow inside a volume is considered to be the sum of the flow of the subnetworks present in the image volume and independent from each other. More precisely:

(4) $${\text{k}}_{\mathrm{ij}}\left(\text{I}\right)=\sum_{\mathrm{cc}=1,\ldots ,m}{\text{w}}_{\mathrm{ij}}\left(\text{cc}\right){\text{k}}_{\mathrm{ij}}\left(\text{cc}\right),$$

where *k*_*ij*_ (*cc*) stands for the permeability tensor element *i*,* j*=1, 2, 3 of the subnetwork *cc* and *m* is the number of all subnetworks that compose the image volume *I*. The weights *w*_*ij*_ (*cc*) are defined as the ratio of the surface of the volume containing the subnetwork *cc* and the surface of the volume covered by all subnetworks. Please note that for the calculation of the latter, we have to correct for overlapping areas among the subnetworks. Thus, *w*_*ij*_ (*cc*) is given by:

(5) $$\begin{array}{cc} {\text{w}}_{\mathrm{ij}}\left(\text{cc}\right) & =\frac{{\text{S}}_{j}\left(\text{cc}\right)}{\sum_{\mathrm{cc}=1,\ldots ,m}{\text{S}}_{j}\left(\text{cc}\right)-\sum_{\begin{array}{c} n,k=1,\ldots ,m\\ k>n \end{array}}{\text{S}}_{j}\left(\text{n}\right)\cap {\text{S}}_{j}\left(\text{k}\right)} \end{array},$$

where *S*_*j*_ (*cc*) stands for the outflow surface of the subnetwork *cc* along direction *j*. Please note that *S*_*j*_(*n*) ∩ *S*_*j*_(*k*) represents the overlapping surface between subnetworks *n* and *k*.

Furthermore, deletions of the blind ends might result in extremely oversimplified versions of the network compared with the original, with the volume after the deletion of blind ends *V*_*cc*_ representing only 15% of the original vascular network within the volume occupied by the subnetwork (*V*_*init*_(*cc*)). Using correlation and regression analysis (Data S1), we discovered a statistically significant linear relationship between the permeability tensor and the ratio of the volume of the subnetwork after the elimination of the blind ends and the initial volume $\left(\frac{V\left(\mathrm{cc}\right)}{V_{\mathrm{init}}\left(\mathrm{cc}\right)}\right)$, with the permeability tensor increasing as the percentage increased. To adjust for this dependency, during the fusing step, the permeability tensors of the subnetworks were rescaled according to the resulting volume after blind end elimination. This was done by multiplying the weights of the tensors with the corresponding reverse ratio: $\left(\frac{V\left(\mathrm{cc}\right)}{V_{\mathrm{init}}\left(\mathrm{cc}\right)}\right)$. After the application of this ad hoc scaling factor, the permeability tensors were no longer linearly dependent on $\frac{V\left(\mathrm{cc}\right)}{V_{\mathrm{init}}\left(\mathrm{cc}\right)}$, confirming the appropriateness of the introduced scaling factor to minimize the effect that the reduced volume caused by elimination of the blind ends had on the tensors.

In the case of confocal data, volume orientation compared with heart orientation is lost. Consequently, to compare different images, the direction *i*=1 is considered to be the direction of maximum flow, direction *i*=2 is the direction with medium flow, and *i*=3 is the direction with the lowest flow. To achieve this, elements *k*_11_, *k*_22_, and *k*_33_ are sorted in descending order. The rest of the elements of the tensor are then rearranged accordingly.

**Figure 1:**
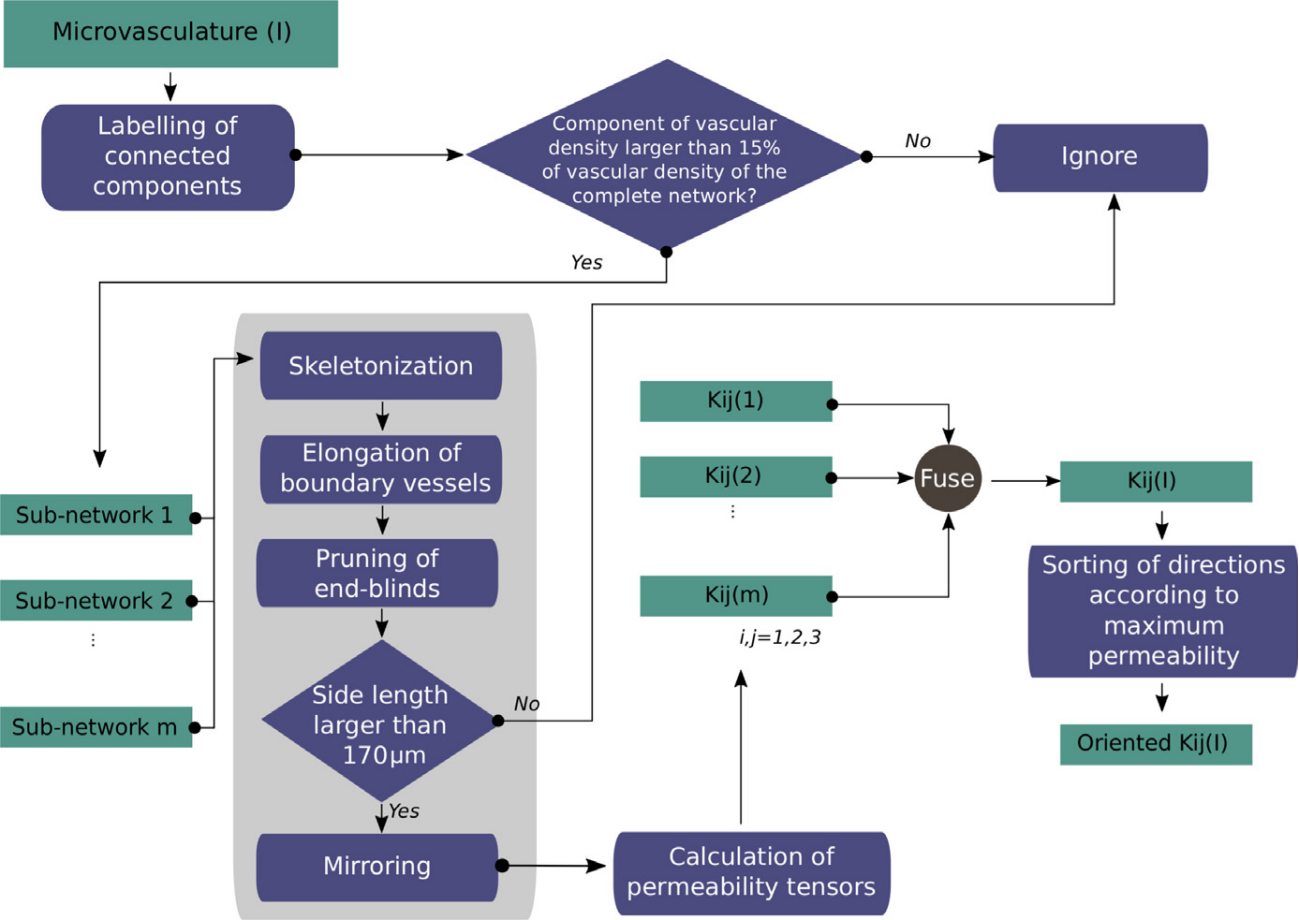
Overview of the proposed pipeline for the calculation of the permeability tensors and myocardial blood flow from microvascular 3‐dimensional anatomical data.

Figure 1 provides an overview of the complete pipeline.

## Results

### Estimated Permeability Tensors Are Close to Physiological Values and Present a Bimodal Pattern in Infarcted Areas in the Aftermath of MI

To understand the changes occurring in the coronary microcirculation after MI, we aimed to quantify the permeability tensors (microvascular conductivity) in the infarcted and remote areas of the pig infarcted hearts and to compare them with those in healthy conditions. Toward this end, we applied the proposed modeling framework to the porcine cardiac volumes described previously.^7^ Images for which the permeability tensor was not computed were excluded from subsequent analysis. Examples of such exclusions include images for which no component represented >15% of the image vascular density or images for which the resulting size of all of its subnetworks was <170 μm. Therefore, the final numbers of images composing our data set were 12 in basal conditions and 11, 12, 9, and 10 for infarcted areas at 1, 3, 7, and 45 days, respectively, after MI. For remote areas, 13, 7, 8, and 12 images were included at 1, 3, 7, and 45 days, respectively, after MI.

**Figure 2:**
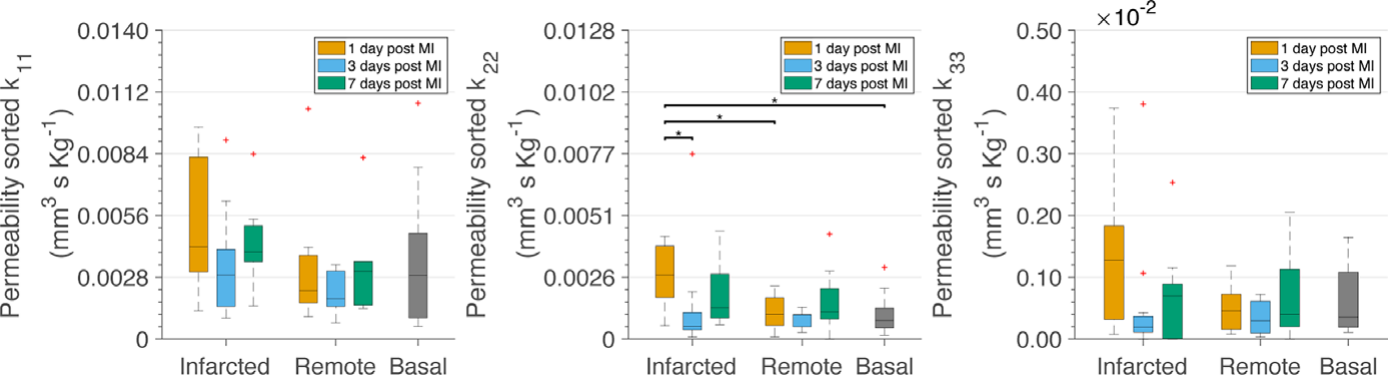
Permeability tensor elements (*k*_11_, *k*_22_, and *k*_33_) after sorting directions by larger permeability. Calculations have been performed considering constant hematocrit of 0.4 in all vessel segments. *P<0.05. The *P* values were calculated with Wilcoxon rank‐sum tests and corrected by the Benjamini–Hochberg procedure for multiple testing. The central lines in the boxes stand for the median, whereas the top and bottom edges of the boxes represent the 25th and 75th percentiles, respectively. The whiskers extend to the most extreme data points that are not considered outliers. The latter are represented using a red cross. The same annotations and statistical hypothesis testing approach apply to all figures in this article. MI indicates myocardial infarction.

In basal conditions, the permeability tensor element along the direction of maximum flow was (3.5 ± 3.2) × 10^−3^ mm^3^/s per kg (Table 1 and Figure 2), within the physiological ranges reported for the rat myocardium, where *k*_11_ was found equal to (3.3 ± 0.8) × 10^−3^ mm^3^/s per kg.^30^ Permeability tensor elements along the direction of medium (*k*_22_) and lower (*k*_33_) flow direction are between less than half of *k*_11_ and an order smaller, confirming a preference of flow along a main direction. The high variability observed among the volumes is expected, taking into account the heterogeneity of the microvasculature and thus of microvascular flow between different regions of the heart.^37^ The off‐diagonal elements of the permeability tensor are practically zero, confirming that cross‐flow is minimal. Moreover, it is worth noting that the direction of maximum flow was the direction along the *z*‐axis in only 1 volume. This is anticipated because the orientation of capillaries in the myocardium is longitudinal and not transversal.

**Table 1:** Changes in Permeability Tensors myocardial blood flow and arteriole‐venule drop in pressure in the Infarcted and Remote Zones of the Heart After MI

Calculated metrics	I1MI	I3MI	I7MI	I45MI	R1MI	R3MI	R7MI	R45MI	Basal
*k*_11_, mm^3^/s/kg	0.0052±0.003	0.0033±0.0024	0.0043±0.0019	0.0098±0.0068	0.003±0.0025	0.0022±0.001	0.0032±0.0022	0.0061±0.0023	0.0035±0.0032
*k*_22_, mm^3^/s/kg	0.0026±0.0013	0.0013±0.0021	0.0019±0.0014	0.0039±0.0033	0.0011±0.0007	0.0009±0.0004	0.0016±0.0014	0.0024±0.0013	0.001±0.0008
*k*_33_, mm^3^/s/kg	0.0013±0.0012	0.0006±0.0011	0.0007±0.0008	0.0014±0.0016	0.0005±0.0004	0.0004±0.0003	0.0007±0.0008	0.0013±0.0008	0.0006±0.0005
MBF *k*_11_, mL/min/100 g	295.72±167.16	186.66±133.4	242.59±107.91	550.63±383.62	169.19±139.06	121.7±56.24	182.31±124.75	341.27±130.64	197.66±179.11
MBF *k*_22_, (mL/min/100 g)	149.3±74.15	71.42±117.29	106.86±78.7	219.27±188.57	63.78±39.05	48.16±21.97	87.65±77.65	133.04±74.75	57.07±45.98
MBF *k*_33_, (mL/min/100 g)	73.06±66.09	31.17±59.56	39.4±45.2	78.38±91.84	27.28±20.63	19.81±15.6	38.69±43.52	72.4±47.7	34.54±28.93
DP, mm Hg	7.16±4.13	16.94±10.81	5.1±2.83	2.85±1.84	23.18±12.86	21.31±13.36	20.41±11.78	9.88±4.27	26.31±25.9
MBF_*k*11_^DP^, mL/min/100 g	77.12±47.4	88.21±60.1	31.79±13.26	30.95±16.51	111.01±138.75	62.74±38.07	121.54±99.22	114.14±52.03	215.2±205.92
MBF_*k*22_^DP^, mL/min/100 g	39.38±22.66	31±42.28	13.98±9.12	13.95±11.88	40.81±29.82	25.21±14.52	60.02±60.2	45.33±30.11	59.5±56.24
MBF_*k*33_^DP^, mL/min/100 g	18.55±18.05	11.96±17.28	5.6±5.74	4.97±5.41	17.96±17.36	11.31±9.66	27.28±34.25	24.17±16.7	36.1±33.38

Data are shown as mean±SD of diagonal elements of permeability tensors (*k*_11_, *k*_22_, and *k*_33_); MBF according to *k*_11_, *k*_22_, and *k*_33_ and constant AV DP (MBF_*k*11_, MBF_*k*22_, and MBF_*k*33_); AV DP; and MBF using the calculated varying DP shown in row 7 (MBF_*k*11_^DP^, MBF _*k*22_^DP^, and MBF _*k*33_^DP^). The AV path is assumed equal to 512 μm. I1MI, I3MI, I7MI, and I45MI indicate infarcted areas at 1, 3, 7, and 45 days, respectively, after MI. Similarly, R1MI, R3MI, R7MI, and R45MI indicate remote areas at 1, 3, 7, and 45 days, respectively, after MI. AV indicates arteriole–venule; DP, drop in pressure. MBF, myocardial blood flow; MI, myocardial infarction.

We then analyzed the changes that occur in the permeability tensors, and thus in the microvascular conductivity, in the infarcted and remote zones of the heart after MI (Table 1 and Figure 2). Of note, there was a bimodal increase of the permeability tensor elements compared with the basal condition and with the corresponding remote area along all directions at infarcted areas 1 and 7 days after MI. For the *k*_22_ element (medium‐flow direction) at day 1 after MI, the differences become statistically significant. This unexpected increased microvascular conductivity at the infarcted areas on day 7 could be related to the high dependence of the permeability tensors on vessel diameter because vessels are enlarged at that time point.^7^ Permeability tensors at infarcted tissue 3 days after MI, and in the remote area, were restored compared with days 1 and 7 and close to values in the basal condition, in accordance with their similar microvascular architecture.^7^

For comparison, Table 2 contains blood flow properties described in this and in following sections when calculated without the application of the scaling factor to account for the simplification of the network after deletion of blind ends. The results are similar to those with the application of a scaling factor (Table 1) but with slightly lower values for the permeability tensors and MBF and higher for AV DP. 

**Table 2:** Blood Flow Properties Calculated Without the Application of the Scaling Factor for Simplification of the Network After Deletion of Blind Ends

Calculated metrics	I1MI	I3MI	I7MI	I45MI	R1MI	R3MI	R7MI	R45MI	Basal
*k*_11_, mm^3^/s/kg	0.0037±0.0023	0.0018±0.0012	0.0022±0.0009	0.0038±0.002	0.0017±0.0021	0.001±0.0006	0.0021±0.0017	0.004±0.0018	0.0028±0.0027
*k*_22_, mm^3^/s/kg	0.0019±0.0011	0.0006±0.0009	0.0009±0.0006	0.0017±0.0014	0.0006±0.0004	0.0004±0.0002	0.001±0.001	0.0016±0.0011	0.0008±0.0007
*k*_33_, mm^3^/s/kg	0.0009±0.0009	0.0002±0.0004	0.0004±0.0004	0.0006±0.0007	0.0003±0.0003	0.0002±0.0002	0.0005±0.0006	0.0008±0.0006	0.0005±0.0004
MBF *k*_11_, mL/min/100 g	210.04±129.1	101.54±69.18	121.55±50.69	211.79±112.97	93.38±116.72	57.41±34.83	116.12±94.79	225.27±102.69	159.5±152.62
MBF *k*_22_, mL/min/100 g	107.25±61.7	35.68±48.67	53.46±34.88	95.48±81.25	34.33±25.08	23.07±13.29	57.34±57.52	89.47±59.42	44.1±41.68
MBF *k*_33_, mL/min/100 g	50.52±49.15	13.77±19.89	21.41±21.93	34.02±37.01	15.11±14.61	10.35±8.84	26.06±32.72	47.71±32.96	26.75±24.74
DP, mm Hg	12.09±11.06	31.18±19.23	10.35±6.06	6.77±4.27	54.05±30.46	50.2±34.12	39.43±31.56	16.24±9.12	44.99±52.12
MBF_*k*11_^DP^, mL/min/100 g	130.22±80.04	162.36±110.61	64.51±26.9	73.53±39.22	258.84±323.52	147.79±89.68	234.8±191.68	187.61±85.53	368±352.12
MBF_*k*22_^DP^, mL/min/100 g	66.5±38.26	57.05±77.82	28.37±18.51	33.15±28.21	95.15±69.53	59.39±34.21	115.95±116.3	74.51±49.49	101.75±96.17
MBF_*k*33_^DP^, mL/min/100 g	31.32±30.47	22.02±31.81	11.36±11.64	11.81±12.85	41.87±40.48	26.65±22.77	52.69±66.17	39.73±27.45	61.72±57.07

Data shown as mean±SD (standard deviation) of diagonal elements of permeability tensors (*k*_11_, *k*_22_, and *k*_33_); MBF according to *k*_11_, *k*_22_, and *k*_33_ and constant AV DP (MBF_*k*11_, MBF_*k*22_, and MBF_*k*33_); AV DP; and MBF using the calculated varying DP in row 7 (MBF_*k*11_^DP^, MBF_*k*22_^DP^, and MBF_*k*33_^DP^) when the scaling factor to account for oversimplification of the network is not applied. The AV path is assumed equal to 512 μm. I1MI, I3MI, I7MI, and I45MI indicate infarcted areas at 1, 3, 7. and 45 days, respectively, after MI. Similarly, R1MI, R3MI, R7MI, and R45MI indicate remote areas at 1, 3, 7, and 45 days, respectively, after MI. AV indicates arteriole–venule; DP, drop in pressure. MBF, myocardial blood flow; MI, myocardial infarction.

It should also be noted that in this work, we assumed constant hematocrit equal to 0.4 in all microvessels. It is, however, a fact that because of the uneven splitting of red blood cells in branching points, the hematocrit is not uniform in all segments of microvascular networks. It is rather heterogeneous, with higher values in daughter vessels with higher flow rates and lower values in those with lower flow rates. This effect is known as *phase separation effect* or *plasma skimming*. To take it into account, Pries and Secomb^38^ established equations that allow calculation of the varying hematocrit of each microvessel segment to be subsequently used for calculating segment viscosity. Based on those equations and an iterative procedure in the calculation of the tensors for each subnetwork (Data S1 and Figure S2), we incorporated varying hematocrit into the calculation of the permeability tensors. When using this approach, the diagonal elements of the permeability tensor *k*_11_, *k*_22_, and *k*_33_ decreased by 23.51±49.22%, 24.89±44.71%, and 40.77±20.59%. This result is in line with earlier studies that have found an effect of varying hematocrit incorporation on blood flow rates of 20%.^39^

### Permeability Tensor–Based Calculation of AV DP and MBF Shows Dynamic Pressure and Flow Alterations After MI

**Figure 3:**
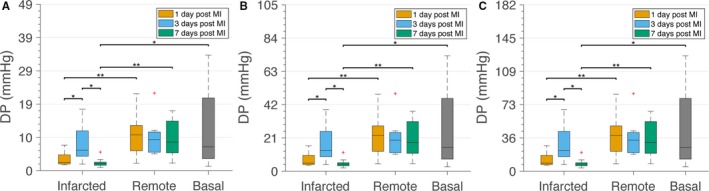
Altered arteriole–venule (AV) drop in pressure (DP) after myocardial infarction (MI). DP was calculated using the larger element of the permeability tensor and different AV path length. DP for a path length 349 μm (**A**), 512 μm (**B**) and 675 μm (**C**). *P<0.05. **P<0.01.

We next sought to calculate the MBF in the microvasculature based on the permeability tensor values obtained. We calculated the AV DP using the MBF values measured in each subject by MRI (Table S1). For that, given the path length equal to 512±163 μm described previously for the pig,^40^ and considering that the path length has an important effect on AV DP, we estimated the DP for 3 possible AV path lengths: (1) 349 μm (mean−SD); (2) 512 μm (mean); and (3) 675 μm (mean+SD; Figure 3). For the subject for which we lacked MBF data by MRI on day 3 after MI, we used the value from the other subject at the same time point. Because of the lack of data regarding changes of the AV path length after MI, we assumed that path length between the AV domain of a network remains unchanged. We found that estimated DPs in remote areas were similar to DPs in healthy tissue. Remarkably, DPs in infarcted areas 1 and 7 days after MI were very low and significantly reduced compared with DPs in healthy conditions (Figure 3). Although previously unnoticed, these results demonstrate that AV DP is impaired after MI. DP on day 3 after MI was closer to the estimated values for volumes in basal conditions, pointing to a possible normalization effect at this specific time point, also observed for other structural microvasculature‐related parameters.

**Figure 4:**
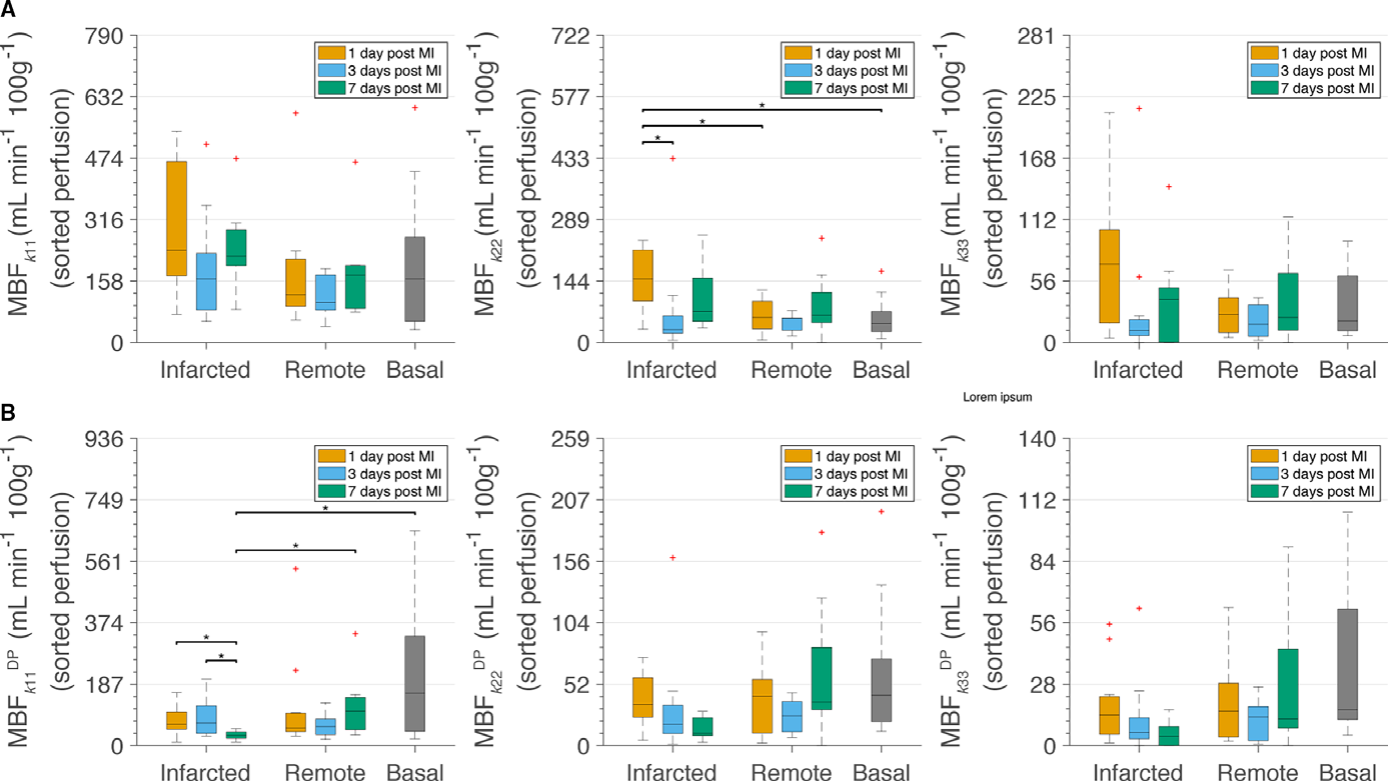
Myocardial blood flow (MBF) calculated using sorted *k*_11_ (MBF_*k*11_/MBF_*k*11_ ^DP^), *k*_22_ (MBF_*k*22_/MBF_*k*22_ ^DP^), or *k*_33_ (MBF_*k*33_/MBF_*k*33_ ^DP^). **A,** MBF considering constant arteriole–venule (AV) drop in pressure (DP) of 19.5 mm Hg and a path length of 512 μm independent of the tissue condition. **B,** MBF considering varying AV DP according to the tissue condition and a path length of 512 μm. The DPs for these simulations are the mean values presented in Table 1. In both cases, calculations have been performed considering constant hematocrit of 0.4 in all vessel segments. *P<0.05. MI indicates myocardial infarction.

To our knowledge, changes in AV DP after MI were not previously reported. We subsequently used the permeability tensor values to calculate the MBF in 2 different contexts: (1) assuming a constant physiological DP of 19.5 mm Hg and (2) incorporating the varying DPs after MI that we observed (Figure 3B). Assuming a physiological and constant DP in all tissue conditions, we noticed a bimodal higher MBF on days 1 and 7 in infarcted areas compared with MBF in basal conditions or in the corresponding remote zone (Figure 4A). We then calculated the MBF using the mean DP values estimated previously, which varied over time, and tissue condition. In this case, infarcted areas 7 days after MI have statistically significant lower MBF (as calculated using *k*_11_) than the healthy, the corresponding remote, and the infarcted areas at days 1 and 3 (Figure 4B). A similar trend was obtained when using *k*_22_ and *k*_33_ to calculate MBF.

We also analyzed these microcirculation‐related parameters at later time points after MI. Permeability tensors calculated 45 days after MI were even higher than the ones calculated for volumes 7 days after MI, DP was even further reduced compared with day 7, and MBF calculated with physiological DP increased but remained reduced with decreased DP. The results are presented in Table 1 and likely reflect the strong remodeling of the microvascular networks with even larger diameters and more disconnections that occurred at this late time point compared with basal microvascular networks.^7^

Findings regarding permeability tensors, AV DP, and MBF at the different tissue areas and time points after MI are summarized in Figure S3.

### Simulations Unravel the Distinct Impact of Vasodilation, Vasoconstriction, and Pruning on Microvascular Conductivity

Because the calculation of the permeability tensors allows us to estimate MBF, we next investigated the impact that different forms of vascular remodeling, and thus possible therapeutic interventions, might have on permeability tensors. The following case scenarios were simulated: (1) dilation of all microvessels by incrementing their diameter by 10%, 20%, and 30%; (2) dilation of a percentage of 10%, 20%, and 30% of the microvessels by incrementing their diameter by 30%; (3) constriction of all microvessels by decreasing their diameter by 10%, 20%, and 30%; (4) constriction of a percentage of 10%, 20%, and 30% of the microvessels by decreasing their diameter by 20%; and (5) pruning of 10%, 20%, and 30% of the microvessels with the smallest radii. In cases 1 and 4, the vessels that remodeled were randomly chosen.

We used volumes under basal conditions and applied the aforementioned remodeling scenarios to the major subnetwork of the image. We subsequently calculated the permeability tensor for the subnetwork for the different remodeling scenarios and compared the result with the tensor obtained without applying remodeling. The mean percentages of change of the diagonal elements of the permeability tensor for all images are provided in Table 3. In addition, Figure S4 provides a graphical overview of the percentage of change in *k*_11_ for every image under basal conditions separately and the mean among them.

**Table 3:** Change of the Permeability Tensor Diagonal Elements Based on Simulations of Different Vascular Remodeling Scenarios Using Tissue Under Basal Conditions

Simulated condition	*k*_11_	*k*_22_	*k*_33_
Dilation of all vessels			
Dilation by 10%	99.64±117.77%	159.24±384.3%	73.1±43.29%
Dilation by 20%	520.41±441.23%	704.54±1300.18%	363.46±128.96%
Dilation by 30%	1819.27±1356.5%	2304.74±3732.15%	1264.64±419.89%
30% increase of randomly selected vessels			
Dilation of 10% of vessels	43.8±71.88%	85.61±257.62%	33.39±32.69%
Dilation of 20% of vessels	144.06±120.81%	228.87±502.1%	107.14±55.8%
Dilation of 30% of vessels	252.33±170.04%	366.82±671.26%	196.15±85.2%
Constriction of all vessels			
Constriction by 10%	−24.98±33.76%	−1.35±141.88%	−26.2±17.82%
Constriction by 20%	−67.34±12.77%	−50.4±85.2%	−67.1±8.78%
Constriction by 30%	−95.78±1.66%	−94.12±8.88%	−95.63±1.47%
20% decrease in diameter of randomly selected vessels			
Constriction of 10% of vessels	15.57±62.18%	67.9±247.79%	12.93±26.15%
Constriction of 20% of vessels	40.48±108.09%	113.06±367.61%	26.09±26.28%
Constriction of 30% of vessels	14.35±69.97%	81.8±292.1%	7.93±26.1%
Pruning of a percentage of the smallest microvessels			
Pruning of 10% of vessels	1.4±101.63%	144.59±348.61%	44.22±100.56%
Pruning of 20% of vessels	106.77±286.96%	239.88±577.49%	56.26±106.24%
Pruning of 30% of vessels	517.65±990.66%	240.04±467.5%	48.99±161.30%

Data shown as mean±SD of the percentage of change of the permeability tensor diagonal elements *k*_11_, *k*_22_, and *k*_33_ based on simulations of different vascular remodeling scenarios using tissue under basal conditions: (1) dilation of all microvessels comprising the microvasculature by incrementing their diameter by 10%, 20%, and 30%; (2) dilation of the 10%, 20%, and 30% of microvessels by increasing their diameter by 30%; (3) constriction of all microvessels comprising the microvasculature by incrementing their diameter by 10%, 20%, and 30%; (4) constriction of the 10%, 20%, and 30% of microvessels by decreasing their diameters by 20%; and (5) pruning of the 10%, 20%, and 30% of the smallest microvessels of the microvasculature.

Dilation of all vessels of the network by only 10% produced a major increase in the elements of the permeability tensor. More precisely, increases of 99.64±117.77%, 159.24±384.30%, and 73.10±43.29% were observed for *k*_11_, *k*_22_, and *k*_33_, respectively. An additional increase of the diameters by 10% results in increases for *k*_11_, *k*_22_, and *k*_33_ by 520.41±441.2%, 704.54±1300%, and 363.46±129%, respectively, with the tensors of few images changing order of magnitude and other being close. When the percentage reached 30%, the increase was so high that the tensors of all images changed by an order of magnitude. These results demonstrate that dilation of all coronary microvessels has a major impact on the permeability tensors. When the change is applied to a percentage of vessels, the permeability tensor always increases but does not arrive at a 10‐fold increase, as it does when all vessels are enlarged.

Because the response of microvessels to vasodilation depends inversely on their diameter (ie, smaller vessels dilate more than larger ones),^41^ we performed additional simulations where the percentage of the increase in diameter depends on the initial vessel diameter. More precisely, we assumed that the maximum vasodilation by a 10%, 20%, and 30% increase in vessel diameter was reached for the smallest microvessels (diameter 0.4 μm), whereas the percentage of change in diameter linearly reduced for larger microvessels, reaching zero for the largest microvessels (diameter 20 μm). The results obtained using this diameter‐dependent vasodilation profile were similar to those considering the same response from all vessels independent of their diameter; *k*_11_, *k*_22_, and *k*_33_ increased by 92.8±110.53%, 151.74±366.54%, and 66.75±41.2%, respectively, for 10% peak dilation; by 396.68±370.2%, 541.71±1021.51%, and 265.77±109.5% for 20% peak dilation; and by 1042.64±917.98%, 1310.68±2160.31%, and 675.01±271.37% for 30% peak vasodilation.

Constriction of diameters of all microvessels by 10% resulted in a decrease of the diagonal elements (*k*_11_, *k*_22_, and *k*_33_) by mean percentages of 24.97%, 1.35%, and 26.20%, respectively. Further constriction of the vessels by an additional 10% led to a decrease of the elements by 67.34%, 50.40%, and 67.10%, respectively. When constriction reached 30%, the decrease of the permeability tensor values was ≈100% for all 3 elements. Interestingly, when 20% constriction is applied to a varying percentage of vessels randomly chosen, the direction of the change might be either positive or negative, with the overall trend of change resulting positive. This indicates that there are cases in which constriction in certain vessels could cause an increase in blood flow.

In the case of pruning of a percentage of the smallest vessels of the microvasculature, the results were highly variable, and the direction of the change of the permeability tensors was either positive or negative depending on the angioarchitecture of the network. This implies that by pruning either effect (ie, increase or decrease of the tensors, depending on the location and the pruned vessel) could be attained. Nonetheless, the mean values of percentage of change were positive. For instance, pruning of 10% of the microvessels resulted in increases for *k*_11_, *k*_22_, and *k*_33_ equal to 1.40±101.63%, 144.59±348.61%, and 44.22±100.56%, respectively. Pruning of 20% of the microvessels produced 106.77±286.96%, 239.88±577.49%, and 56.26±106.24% respective increases to the diagonal elements. When 30% of the smallest microvessels were pruned, the tensor diagonal elements increased by 517.65±990.66%, 240.04±467.50%, and 48.99±161.30%, respectively.

**Figure 5:**
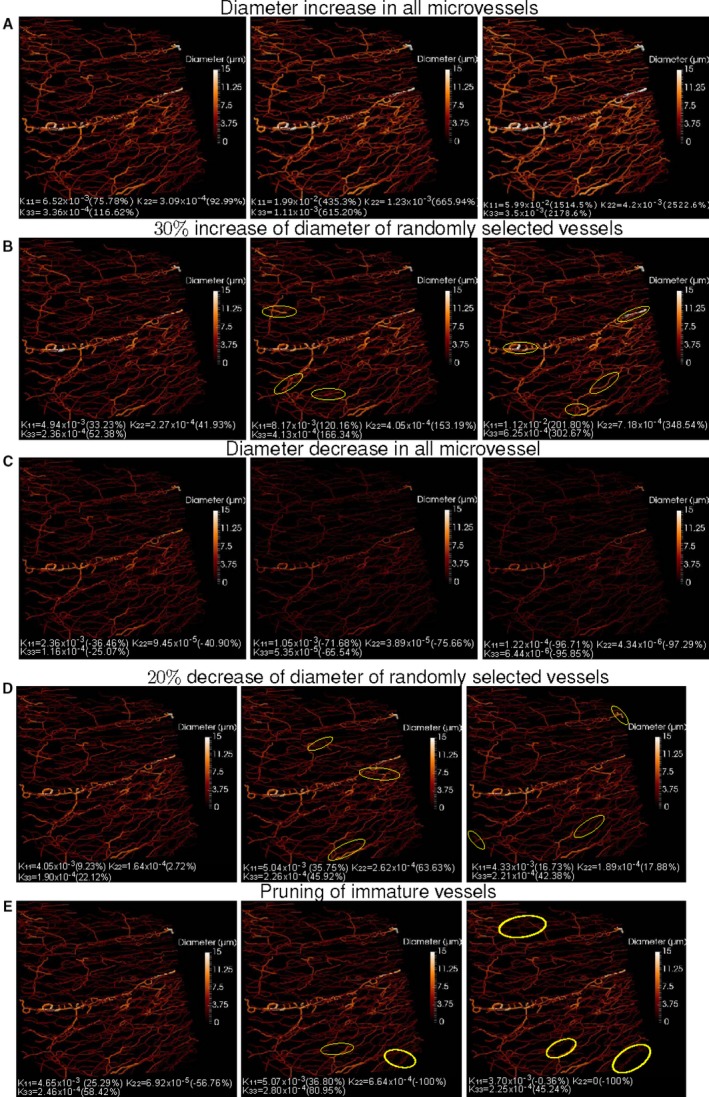
Example with modified radii and number of segments used in the study of the effect of vessel dilation, constriction, and pruning on the permeability tensors. **A**, From left to right, increase by 10%, 20%, and 30% of vessel diameter of all microvessels that compose the microvasculature. **B**, 30% increase of vessel diameter applied to the 10%, 20%, and 30% of vessels. **C**, Decrease of vessel diameter of all microvessels. (**D**) Decrease of vessel diameter by percentage of microvessels: 10%, 20%, and 30% (from left to right). **E**, Pruning of 10%, 20%, and 30% of the capillaries with the smallest radius. The diagonal permeability tensor elements are provided Below each image. In parentheses, the percentages of decrease (negative sign) or increase (positive sign) with respect to the original values (ie, without any remodeling). Image corresponds to tissue from basal conditions. Some examples of remodeled vessels (**B**,** D**) or areas of pruned vessels (**E**) are highlighted in yellow circles.

Figure 5 provides an example following remodeling on the basis of the aforementioned scenarios and the resulting diagonal elements of the permeability tensor. Before any remodeling taking place, *k*_11_, *k*_22_, and *k*_33_ were 3.7×10^−3^, 1.59×10^−4^, and 1.55×10^−4^ mm^3^/s per kg, respectively.

We next sought to mimic possible therapies by simulating vascular remodeling strategies involving vasodilation, vasoconstriction, or pruning using images of tissue on day 3 after MI, which is the time point we identified as better suited for angiointerventions. Mean and standard deviation of percentage change in *k*_11_, *k*_22_, and *k*_33_ and for images of infarcted or remote areas, respectively, are summarized in Table 4. In addition, Figure S5 provides a graphical representation of the percentage of change in *k*_11_ for every image of each region, caused by the different modes of vascular remodeling. The results indicate similar trends in response to the different modes of vascular remodeling 3 days after MI, as observed for basal tissue. Nonetheless, the microvasculature of basal and remote areas on day 3 after MI present greater similarity in their response, whereas the impact of dilation and pruning on *k*_11_, is notably higher for infarcted volumes than for volumes of the other 2 areas. Moreover, the fact that even after infarction, pruning and constriction might not necessarily cause a decrease in the tensor but, on the contrary, can increase permeability tensors and therefore MBF—a fact that can be related to the Braess Paradox^42^—suggests that modulating vascular tone in any direction after MI may constitute a new therapeutic opportunity in the future.

**Table 4:** Percentage of Change of the Permeability Tensor Diagonal Elements Based on Simulations of Different Vascular Remodeling Scenarios Using Tissue From Remote and Infarcted Areas 3 Days After MI

Simulated condition	k_11_	k_22_	k_33_
Dilation by 10%			
R	221.82±335.91%	1256.52±3009.25%	117.91±144.76%
I	419.58±840.58%	68.49±120.82%	58.66±44.75%
Dilation by 20%			
R	855.61±1028.56%	3227.07±7254.17%	488.43±338.87%
I	1572.26±2741.23%	421.26±443.4%	336.48±135.86%
Dilation by 30%			
R	2872.93±3431.13%	9532.58±20954.24%	1655.98±1017.35%
I	6112.94±12142.87%	1517.67±1443.88%	1226.69±453.55%
30% Increase of randomly selected vessels			
Dilation of 10% of vessels			
R	134.28±246.51%	896.56±2228%	52.01±108.09%
I	218.94±472.05%	18.77±91.87%	11.91±32.06%
Dilation of 20% of vessels			
R	269.19±381.23%	1164.63±2778.21%	130.21±144.09%
I	474.79±767.15%	84.38±160.85%	77.33±57.51%
Dilation of 30% of vessels			
R	436.54±640.34%	1386.63±3216.4%	218.35±176.53%
I	845.82±1444.27%	147.62±201.8%	158.14±99.19%
Constriction of all vessels			
Constriction by 10%			
R	27.75±147.8%	467.38±1278.1%	−14.86±62.32%
I	73.61±252.64%	−42.78±34.87%	−37.42±19.62%
Constriction by 20%			
R	−39.8±80.46%	138.58±540.41%	−65.03±24.25%
I	−34±86.44%	−78.97±11.22%	−71.94±11.28%
Constriction by 30%			
R	−92.17±11.82%	−70.31±67.61%	−95.79±3.16%
I	−92.6±9.76%	−97.7±1.28%	−96.11±2.24%
20% Decrease in diameter of randomly selected vessels			
Constriction of 10% of vessels			
R	99.25±233.9%	795.81±2012.64%	29.67±96.25%
I	96.38±245.28%	−7.41±68%	−9.27±25.65%
Constriction of 20% of vessels			
R	158.53±327.89%	836.69±2088.48%	50.41±101.03%
I	139.02±329.07%	−1.47±81.02%	2.15±32.98%
Constriction of 30% of vessels			
R	148.25±356.85%	510.46±1355.81%	26.89±93.54%
I	95.78±294.8%	−27.74±63.89%	−5.23±32.51%
Pruning of a percentage of the smallest microvessels			
Pruning of 10% of vessels			
R	180.88±278.14%	1221.15±2947.87%	47.2±117.52%
I	632.55±1843.79%	61.6±172.81%	−3.25±31.72%
Pruning of 20% of vessels			
R	325.39±362.3%	1274.65±2824.18%	69.29±166.74%
I	1419.43±3706.85%	106.78±185.98%	33.78±73.88%
Pruning of 30% of vessels			
R	513.62±598.17%	1543.78±2669.19%	985.77±2418.45%
I	682.92±1779.71%	22.19±141.59%	45.01±68.76%

Data shown as mean±SD of the percentage of change of the permeability tensor diagonal elements *k*_11_, *k*_22_, and *k*_33_ based on simulations of different vascular remodeling scenarios using tissue from remote and infarcted areas 3 days after MI: (1) dilation of all microvessels comprising the microvasculature by incrementing their diameter by 10%, 20%, and 30%; (2) dilation of the 10%, 20%, and 30% of microvessels by increasing their diameter by 30%; (3) constriction of all microvessels comprising the microvasculature by incrementing their diameter by 10%, 20%, and 30%; (4) constriction of the 10%, 20%, and 30% of microvessels by decreasing their diameters by 20%; and (5) pruning of the 10%, 20%, and 30% of the smallest microvessels of the microvasculature. Rows marked with R and I correspond to results from remote areas and infarcted areas, respectively. MI indicates myocardial infarction.

## Discussion

In this article, we present the first study of microvascular perfusion based on 3D detailed anatomical data of the pig cardiac capillary bed spanning several stages after MI and compared with healthy conditions. For the purposes of the study, we developed an image‐based framework that permitted us to adopt a CFM to perform the simulations. We obtained permeability tensors for the healthy cardiac tissue of the pig that were close to values estimated for the rat myocardium, where *k*_11_ was found equal to (3.3±0.9)×10^−3^ mm^3^/s per kg,^30^ reinforcing the validity of our approach. Smaller values have been, however, calculated for the brain (4.28×10^−4^ 25). After MI, we discovered a bimodal increase in permeability tensor values for all diagonal elements at days 1 and 7. This increase correlates with the altered angioarchitecture of the network and, particularly, with the enlarged vessel diameters. It is interesting to highlight that a bimodal pattern has also been described for myocardial edema at similar time points after MI. The increased vascular vasodilation and plasma leakage after reperfusion, along with the remodeling of the matrix and tissue during healing, seem to account for the early and late edema waves, respectively.^43^ It is therefore tempting to speculate that these events could also contribute to the bimodal increase in the permeability tensors (microvascular conductivity) observed for the microvasculature, consisting of enlarged microvessels at days 1 and 7 after MI. On the contrary, permeability tensor values for the constricted vessels on day 3 after MI were restored and close to values for healthy tissue.

Notably, MBF in infarcted areas 7 days after MI was slightly increased if calculated using physiological AV DP. Nonreduced blood flow 7 days after MI contradicted our findings by means of succolarity.^7^ Succolarity encapsulates fluid capacity to flow within a network and was found to be reduced in infarcted areas 7 days after MI. This unexpected difference could be related to the fact that during calculation of succolarity, blood rheological properties and the strong dependence of blood flow on diameter are not taken into account. However, it is also possible that it is related to the fact that the DP used to calculate MBF was not physiologically accurate. To our knowledge, no previous reports have provided measurements of AV DP in the post‐MI microvasculature; therefore, our work identifies this reduced DP as an additional altered parameter that can contribute to the pathogenesis and progression of post‐MI damage. Thus, when DP was adjusted to the time point and area after MI, we found that MBF 7 days after MI would decrease compared with basal areas or the corresponding remote areas, as expected and in accordance with our succolarity data. Without measurements of blood pressure, quantification of changes in MBF should be carefully made. Unfortunately, a lack of topological information and measurements at voxel level in our MRI data did not permit direct validation of the approach and subsequent necessary adjustments. However, literature values regarding MBF, although variable, show that the estimated MBF values by means of our approach (Table 1) are generally close to reported values. Moreover, earlier studies on MBF confirm that high variability and different trends after MI should be anticipated, depending on the transmural layer (ie, myocardium to epicardium), the anterior or posterior position, and the level from base to apex of the heart.^37^, ^44^ Nevertheless, our novel observations on dynamic reduction of AV DP after MI suggest that MBF could be improved with therapies able to preserve DP close to physiological levels.

In a recent study, the MBF of pigs was estimated using microspheres.^45^ Mean±SD for MBF was found to be 122±92 and 99±31 mL/min per 100 g using 1.5‐ and 3‐T MRI, respectively, during rest. The corresponding values during hyperemia were 221±167 and 208±81 mL/min per 100 g, whereas during reduced flow (half‐flow), they were 56±37 and 55±26 mL/min per 100 g. It is worth noting that similar values could be calculated with contrast‐enhanced cardiac MRI. Camici et al^46^ summarized data from photon emission tomography studies of healthy humans, with the highest reported MBF values being 120±30 mL/min per 100 g at baseline and 440±90 mL/min per 100 g during hyperemia. In an effort to reduce variability in MBF reported in literature, Chareonthaitawee et al^47^ used a large cohort of human volunteers to measure MBF. Mean±SD for baseline MBF corrected for workload was calculated as 133±31.6 mL/min per 100 g, with values ranging from 73.6 to 242.8. Using the dog animal model, a study with microspheres injected at 15 seconds, 15 minutes, 4 hours, and 3 days after 2‐hour coronary occlusion and reperfusion and with control subjects demonstrated that immediately after reperfusion, the MBF was increased and even higher than in noninfarcted subjects but was reduced 3 days after MI.^37^ Moreover, there was high variability in the estimated values depending on the region from the epicardium toward myocardium. In the same animal model, a study of 5‐minute repetitive occlusion and subsequent reperfusion found that at 1 week after MI, the MBF returned to normal levels in both infarcted and remote areas.^48^ It is worth noting that increases in blood flow after occlusions so as to reach the initial levels can be explained by the autoregulatory mechanisms of the heart that permit maintenance of blood flow as constant despite changes in perfusion pressure, such as those caused by occlusions.^41^

Our simulations of vascular remodeling also provide interesting data of value for possible clinical applications. Our findings point to vasodilatation of all microvessels as a strategy that could help increase microvascular conductivity and thus MBF after MI. The effect of vasodilators such as adenosine have already been tried in MI animal models and in patients, with limited success. This may be related to the poor drug response of vascular smooth muscle cells,^49^ and our data suggest that passive vasodilation by, for example, expanding the blood volume may be more attractive. Unexpectedly, our data also show cases in which constriction in certain microvessels could cause an increase in blood flow. Further work will be required to understand the nature of the best microvessels to be constricted and the means by which to achieve this goal. Increase or decrease of the tensors was achieved depending on the location and the pruned vessel. These results suggest that refined pharmacological interventions aiming to vasodilate, vasoconstrict, or prune certain microvessels may constitute novel therapeutic strategies to improve MBF and thus reduce harmful consequences of MI.

Some limitations of this work could be addressed by future research. Given the challenging nature of the task of simulating perfusion with anatomical data and the particular nature of our relatively small volumes, several simplifications were necessary in postprocessing of the volumes to convert the initial microvascular data into fully connected networks without blind ends. Nonetheless, blind ends might affect the calculation of the tensors and perfusion. In earlier work regarding the tumor network, blind ends were found to affect the calculated perfusion values but with variation due to the different percentage of blind ends taken into account remaining within the experimentally reported values.^13^ Consequently, although our assumption of a minor contribution of blind ends probably represents the reality, it would be interesting to study the impact of blind ends on the calculation of permeability tensors and perfusion if data on pressure or blood flow on blind ends were to become available. Another important limitation of the present study is that opportunities for validation are limited. It would be of particular interest to study the relation of the presented results with measurements from MRI. However, to achieve this, information regarding volume topology would be required, along with voxel‐level measurements from MRI. In addition, the voxel size of a typical MRI is on the order of millimeter or submillimeter, which means that resolution would not be high enough to resolve volumes of 300 μm length. Nonetheless, it might be possible for future studies to apply the developed framework to obtain insights into the relation between blood flow at micro‐ and macroscales by uniting several sequential volumes. This would be of paramount importance in clinics because it could lead to translation of measurements at organ scale to conclusions regarding alterations of the microvasculature, a major determinant of the outcome after successful reperfusion of the epicardial arteries. Last, high variability in the estimated values and the small number of samples after exclusions of several images of our data set reduced the power of statistics, and possible statistically significant differences might not have been detected.

This work marks the first effort to simulate tissue‐scale properties of blood flow using anatomical data directly rather than idealized or statistically generated data. We used data from basal conditions and from different time points after MI and from both infarcted and remote areas from a highly translational animal model. The permeability tensors calculated for the basal condition are within the physiological limits according to previous work. Concomitantly, the estimated AV DP using MBF obtained from MRI points to an altered function of the microvasculature on days 1 and 7 after MI. In addition, according to the data at 45 days after MI, the AV DP does not improve without the application of therapy. Despite the presence of increased blood flow, the remodeling compensatory mechanisms are not adequate to allow efficient oxygenation.

The computational tools developed as part of this work and their application to a porcine MI ischemia–reperfusion model allow for deeper understanding of microvascular alterations in MI by modeling of microcirculation at different stages after injury. To the best of our knowledge, this study is the first in which tissue‐scale blood flow properties have been predicted at different stages following MI and compared with tissue from basal conditions based on real 3D anatomical data with submicrometer resolution and on a continuum homogenization model. The permeability tensors extracted as a result of this work can also be used to parameterize organ‐scale models of the heart at different time points after MI. Future work could include combining the CFM with a discrete model to explore changes taking place in the distribution of hemodynamics—data that are not possible to measure experimentally in individual microvessels with today's imaging systems.

## Sources of Funding

The research leading to these results has received funding from the People Programme (Marie Curie Action) of the European Union's Seventh Framework Programme (FP7/2007–2013) under REA grant Agreement 608027 and from the Spanish Ministerio de Ciencia, Innovación y Universidades (SAF2017‐83229‐R) to Arroyo. The CNIC (Centro Nacional de Investigaciones Cardiovasculares) is supported by the Ministerio de Ciencia, Innovación y Universidades and the Pro CNIC Foundation, and is a Severo Ochoa Center of Excellence (SEV‐2015‐0505). Popel was supported by NIH grant R01HL101200 from the National Heart, Lung and Blood Institute, NHLBI. Santos acknowledges founding from Ministerio de Ciencia, Innovación y Universidades (TEC2015‐66978‐R). El‐Bouri was funded by a Doctoral Training Partnership studentship, grant reference EP/M50659X/1.

## Disclosures

None.

## Supporting information


**Data S1.** Supplemental Methods.
**Table S1.** Characteristics of the Participants in the Study. Table reproduced from Gkontra et al^9^ (Creative Commons license^10^)
**Figure S1.** Mirroring of the original image colour‐coded with light green (7 color scale). 2D slices along x,y and z directions of the 3D resulting mirrored image that con‐tains 8 copies of the original image and periodic boundaries on opposites faces.
**Figure S2.** Overview of the approach for incorporating the phase separation effect in the calculation of permeability tensors.
**Figure S3.** Summary of the changes in microvascular conductivity, AV pressure drop and MBF after MI. Dashed lines correspond to remote areas, while solid to infarcted. The scheme highlights the bimodal changes obtained for the per‐meability tensors which increase at day 1 and day 7 after MI with partial restora‐tion at day 3 (closer to basal). MBF calculated assuming physiological AV pressure drop independently of tissue condition, i.e. 19mmHg, follows the bimodal increase of the tensors at infarcted areas (blue‐green line). On the contrary, when condition‐dependent AV pressure drop is used, MBF remains reduced at infarcted areas (purple line).
**Figure S4.** Impact of vascular remodeling strategies on microvascular conductivity obtained by simulations using images of basal conditions. The pseudocolored map represents the impact (ie, percentage of change, %) of *k*_11,_ on the permeability tensors of the simulations for distinct vascular remodeling strategies described at left. Every column corresponds to an image, with the last column representing the mean among all images for the specific tissue condition.
**Figure S5.** Impact of vascular remodeling strategies on the microvascular conductivity obtained by simulations using images on day 3 after myocardial infarction (MI). The pseudocolored map represents the impact (ie, percentage of change, %) of *k*_11_, of the simulations for distinct vascular remodeling strategies described at left. **A**, Change in *k*_11_ when vascular remodeling is simulated using images from remote areas on day 3 after MI. **B**, Change in *k*_11_ when vascular remodeling is simulated for images from infarcted areas on day 3 after MI. Every column corresponds to an image, with the last column representing the mean among all images for the specific tissue condition.
**Figure S6.** Dependency of the permeability tensor on representative volume element. Three different sizes were investigated—256×256×*Nz*, 512×512×*Nz*, and 1024×1024×*Nz* voxel—with *Nz* indicating the size of the image along the *z*‐axis. The original image volume was thus decomposed into 16, 4, and 1 vol/U, respectively, depending on the unit size. Tensors for the subvolumes under investigation were calculated by applying the proposed approach for the calculation of permeability tensors from anatomical data on each subvolume. The asterisk size is proportional to the volume of the units normalized by a volume size of 0.0074 mm^3^, corresponding to 1024×1024 ×50 voxels (0.379, 0.379, 1007 μm). The different asterisk sizes indicate that the resulting connected network inside the unit might be smaller than the initial unit to which the image was decomposed. For each time point, the plots at left show the maximum element of the permeability tensor in relation to volume size of the subnetworks of all images available in our data set. At right, there are plots for every image (1 line/image) after fusing the tensors of the units in which it was decomposed. The permeability tensor in this figure is given as the median of the different subvolumes for simplicity.
