# Supplementary material for: Dynamic Changes in Microvascular Flow Conductivity and Perfusion After Myocardial Infarction Shown by Image‐Based Modeling
Source: J Am Heart Assoc. 2019 Mar 22;8(7):e011058. doi: 10.1161/JAHA.118.011058 (PMC6509718; doi:10.1161/JAHA.118.011058)
Supplement: Supplementary file 1 — Data S1. Supplemental Methods. Table S1. Characteristics of the Participants in the Study. Table reproduced from Gkontra et al9 (Creative Commons license10) Figure S1. Mirroring of the original image colour‐coded with light green (7 color scale). 2D slices along x,y and z directions of the 3D resulting mirrored image that con‐tains 8 copies of the original image and periodic boundaries on opposites faces. Figure S2. Overview of the approach for incorporating the phase separation effect in the calculation of permeability tensors. Figure S3. Summary of the changes in microvascular conductivity, AV pressure drop and MBF after MI. Dashed lines correspond to remote areas, while solid to infarcted. The scheme highlights the bimodal changes obtained for the per‐meability tensors which increase at day 1 and day 7 after MI with partial restora‐tion at day 3 (closer to basal). MBF calculated assuming physiological AV pressure drop independently of tissue condition, i.e. 19mmHg, follows the bimodal increase of the tensors at infarcted areas (blue‐green line). On the contrary, when condition‐dependent AV pressure drop is used, MBF remains reduced at infarcted areas (purple line). Figure S4. Impact of vascular remodeling strategies on microvascular conductivity obtained by simulations using images of basal conditions. The pseudocolored map represents the impact (ie, percentage of change, %) of k 11, on the permeability tensors of the simulations for distinct vascular remodeling strategies described at left. Every column corresponds to an image, with the last column representing the mean among all images for the specific tissue condition. Figure S5. Impact of vascular remodeling strategies on the microvascular conductivity obtained by simulations using images on day 3 after myocardial infarction (MI). The pseudocolored map represents the impact (ie, percentage of change, %) of k 11, of the simulations for distinct vascular remodeling strategies described at left. A, Change in k 1 [file JAH3-8-e011058-s001.pdf]

SUPPLEMENTAL MATERIAL

Data S1.

Supplemental Methods

Permeability tensors calculation based on homogenization

Calculation of permeability tensors was performed by applying a continuous two-phase porous perfusion model that has been adapted from El-Bouri and Payne.[1] Well-separated micro- (microvascular network) and macro- (tissue) length scales are assumed, i.e. the ratio of the length of the volume that contains the microvasculature is significantly smaller than the length of the entire cardiac tissue, as well as non-leaky vessels. At the micro-scale, it is additionally assumed that the tissue consists of spatially periodic micro-cell units that contain two phases; (i) the microvasculature, which permits the blood to flow and, (ii) the interstitium, which consists of cells and extravascular matrix and it behaves as a porous medium. It shlould be noted that periodicity is necessary in order to ignore secular terms. Furthermore, blood flow of the microvessels is considered incompressible and dominated by viscous forces (Stokes flow). Taking these assumptions into account and the fact that volume averaged tissue-scale blood velocity equals the surface mean blood velocity to leading order,[2] the homogenization technique of Shipley and Chapman[3] is applied and the volume averaged form of Darcy's law is deduced to:

$$\mathbf{u} = -\mathbf{K}(I)\nabla p \qquad (1)$$

where $\mathbf{K}(I)$ is the permeability tensor of the image volume I and given the 3D nature of I it is of size $[3 \times 3]$. i.e. equation 1 is written analytically as:

$$\begin{bmatrix} u_1 \\ u_2 \\ u_3 \end{bmatrix} = - \begin{bmatrix} k_{11} & k_{12} & k_{13} \\ k_{21} & k_{22} & k_{23} \\ k_{31} & k_{32} & k_{33} \end{bmatrix} \begin{bmatrix} \nabla p^1 \\ \nabla p^2 \\ \nabla p^3 \end{bmatrix} \qquad (2)$$

where ∇p^i stands for the pressure gradient applied on the tissue along direction i, while u_j in 3D is the surface mean blood velocity component along direction j and it

is thus given by:

$$u_j = Q_j / S_j \tag{3}$$

where S_j is the area of the outflow surface at direction j and Q_j represents the sum of the flow rates of the capillaries on that surface.

By combining equations 2 and 3, the elements of the permeability tensor can be calculated by:

$$k_{ij}(I) = \frac{\sum_{m=1}^{M_j} \mathbf{q}_m^j}{S_j \nabla p^i}, \quad i, j = 1,\ 2,\ 3 \tag{4}$$

where M_j is the number of capillaries on surface j.

Derivation of myocardial blood flow equation

Perfusion can be calculated by integrating the velocity over the cross-sectional area (A) of the material through which flows the fluid, i.e. in this work, the myocardium. If the velocity is parallel to the axis of one of the main directions of flow, then by using the corresponding diagonal element of the permeability tensor (k_{ii} , $i = 1, 2, 3$) which encompasses the microvascular conductivity of the microvasculature feeding the myocardium, perfusion can be calculated by:

$$Perfusion(\mu m^3 s^{-1}) = k_{ii} \times \frac{\Delta p}{l} \times 0.133 \times 10^{-3} \times A \tag{5}$$

where Δp stands for the AV pressure drop in $mmHg$ over a microvascular path of length l given in μm. It should be noted that 0.133×10^{-3} is a conversion factor for pressure units from $mmHg$ to $kg\mu m^{-1}s^{-2}$, while the cross-sectional area over which the velocity is integrated is expressed in μm.

Myocardial blood flow can be expressed:

$$MBF(mL/min/100g) = \frac{Perfusion \times 60 \times 100}{\rho \times A \times l} \tag{6}$$

where ρ represents myocardial density (ρ), while 60 and 100 are conversion factors

from minutes to seconds, and $1g$ to $100g$ respectively.

Taking into account the aforementioned relationships, i.e. the relation between perfusion and the permeability tensors (eq. 5), and the relation between perfusion and MBF (eq. 6),[4] the latter can be calculated using the permeability tensors by:

$$MBF(mL/min/100g) = \frac{k_{ii} \times \Delta p \times 0.133 \times 10^{-3} \times 60 \times 100}{\rho \times l^2} \tag{7}$$

Incorporation of phase separation effect into the calculation of the permeability tensors

We incorporated an iterative procedure in the calculation of the tensors for each sub-network as show in Supplemental Fig. 2.

In brief, a constant hematocrit (0.4) was initially assumed in order to calculate the permeability tensor along the x-direction. The pressure solution that resulted in the calculation of the permeability tensor elements $k_{11},\ k_{12},\ k_{13}$ was used as an input to an iterative publicly available algorithm[5] for the calculation of varying hematocrit when separation effect on junction points is taken into account.[6] It is worth mentioning that the equations regarding apparent viscosity of this implementation, although were derived from data for the rat mesentery, are expressed for human blood parameters.[7] In order to account for differences in blood parameters between different species, the diameters of the vessels should be scaled using the cubic root of the ratio of the volume of red blood cells for humans and the species under investigation. Therefore, here, the diameters of the vessels were scaled by multiplying by $(\frac{V_{human}}{V_{pig}})^{1/3}$, where $V_{human} = 90fl$ and $V_{pig} = 54fl$.[8] Once the solution converges or reaches the maximum number of permitted iterations (100), the permeability tensors are calculated with the new varying values of the hematocrit. Once the new permeability tensors differ less than $2 \times 10^{-17} mm^3\ s\ kg^{-1}$ in all elements from the last iteration, the procedure is considered as converged and the calculated tensor is the final one. As for some volumes did not converge, an additional limit of a maximum of 300 iterations was set.

Calculation of cut-off size of Representative Volume Element (REV)

To define the cut-off side length along the x and y directions below which calculation of permeability tensors could lead to errors, we considered the capillary bed of each volume as the meso-scale which is homogeneous inside smaller areas of itself. More precisely, the volume was divided into smaller microscopic units with a size of $256 \times 256\times$ Nz, $512 \times 512\times$ Nz, $1024 \times 1024\times$ Nz voxels, where Nz stands for the size of the original image I along the z-axis (in voxels). We applied the developed framework at each unit to calculate permeability tensors for each one of them. We concluded that the permeability tensor for volumes below 512 voxels were frequently overestimated, with the larger element of the permeability tensor being an order higher than values reported in literature[4] (Supplementary Fig. 6). Therefore, in our subsequent analysis, we excluded sub-networks whose size was smaller than this cut-off limit. It should be noted that the voxel size of the majority of our images is $[0.379,\ 0.379,\ 1.007]\ \mu m$. Thus, the calculated cut-off limit of 512 voxels along the x and y directions corresponds to a physical size of 194 μm. After inspection of the results for all images, the cut-off size for excluding connected components from subsequent analysis was given a small margin and was set equal to 170 μm in order to avoid excluding connected components with size very close to 194 μm for which the tensors were correctly calculated. In sum, the permeability tensor of a $3D$ image was calculated as the weighted sum of the connected components whose side length along x-y axis was larger than 170 μm prior to application of the mirroring procedure.

Calculation of scaling parameter

We performed correlation and regression analysis and discovered a statistically significant linear relation of the permeability tensor to the ratio of sub-network vascular volume ($V(cc)$) to the vascular volume in the corresponding volume of the image ($V_{init}(cc)$) prior to any post-processing having taken place. More precisely, the

radii and length of all vascular segments, prior and after repetitive elimination of the blind-ends until the microvascular network under investigation is fully connected and has no blind-ends, had already been calculated. Therefore, we used them to calculate the two volumes, i.e. $V(cc)$ and $V_{init}(cc)$, by considering each microvessel as a tube of constant radius. Using the basal condition, we calculated the Kendall's Tau correlation coefficient to study whether there is any association between the permeability tensor and the ratio of volume of the sub-network prior and after the elimination of the blind ends, i.e. $\frac{V(cc)}{V_{init}(cc)}$. The coefficient was equal to 0.47 (p-value$= 0.008$) and 0.66 (p-value $= 8 \times 10^{-5}$) for k_{11} and k_{22} respectively denoting a dependency of the permeability tensor on the ratio. We applied a scaling factor equal to the reverse of the ratio ($\frac{V(cc)}{V_{init}(cc)}$) to the permeability tensors in order to adjust them on the basis of this dependency. The scaling factor was incorporated by multiplying the weights $w_{ij}(cc)$ by it. By applying linear regression analysis before and after application of the scaling factor, we noticed that the permeability tensors were no longer linearly dependent on $\frac{V(cc)}{V_{init}(cc)}$ ($y \sim \beta_0$, $\beta_0 = 4 \times 10^3$ (p-value$= 9.49 \times 10^{-28}$)).

Table S1. Characteristics of the subjects used in the study. Table has been reproduced from Gkontra et al.[9] (Creative Commons license[10]).

Subject	1	2	3	4	5	6	7	8	9	10	11
Time after infarction	Basal	Basal	24 hours	24 hours	3 days	3 days	7 days	7 days	45 days	45 days	45 days
Body weight (Kg)	34	30.50	34	32.50	40	34	47.50	39.50	56.5	53	57
Left ventricle (LV) mass (g)	55.83	60.50	49.95	57.84	71.18	56.65	88.90	60.60	70.89	60.59	56.88
LV end diastolic volume (ml)	82.63	88.00	95.35	117.3	100.08	96.41	117.51	138.51	128.42	134.9	130.47
LV end systolic volume (ml)	43.20	42.70	53	77.87	44.04	59.61	62.22	87.15	83.85	93.9	87.37
Ejection Fraction (%)	47.74	51.40	44.41	33.61	56	38.17	47.05	37.08	34.71	30.39	33.04
Infarct Size (%)	0	0	29	19.17	16.61	31.01	25.38	41.59	33.37	35.41	29.01
Hematocrit (%)	30.2	32.6	31.1	21.8	29.2	28	30.4	29	33.1	32.3	30.4
Blood Flow LW / R * ($ml/min/100g$)	121.9	86.9	135.5	142.5	N/A †	103.50	114.1	159.40	175.5	139.1	134.8
Blood Flow I ‡ ($ml/min/100g$)	-	-	59.7	104.6	N/A †	107.00	58.9	50.90	29.9	79.3	88.1

*LW / R refer to measurements taken from the LV lateral wall (LW) for the control subjects one and two, and to measurements taken from the remote areas (R) for subjects that have suffered infarction (subjects three to eleven).

†N/A: Information not available due to missing magnetic resonance imaging (MRI) sequence

‡I refers to measurements taken from the infarcted areas (I) for subjects that have suffered infarction.

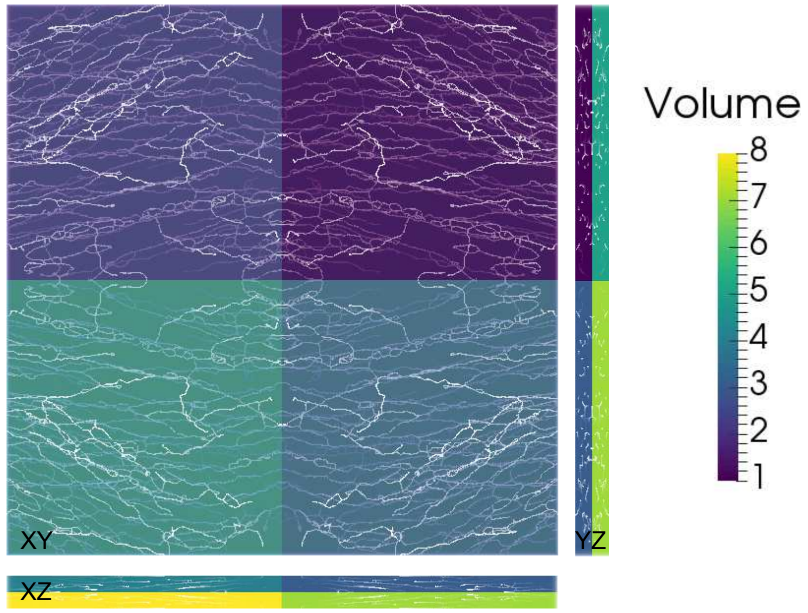

Figure S1. Mirroring of the original image colour-coded with light green (7). 2D slices along x,y and z directions of the 3D resulting mirrored image that con-tains 8 copies of the original image and periodic boundaries on opposites faces.

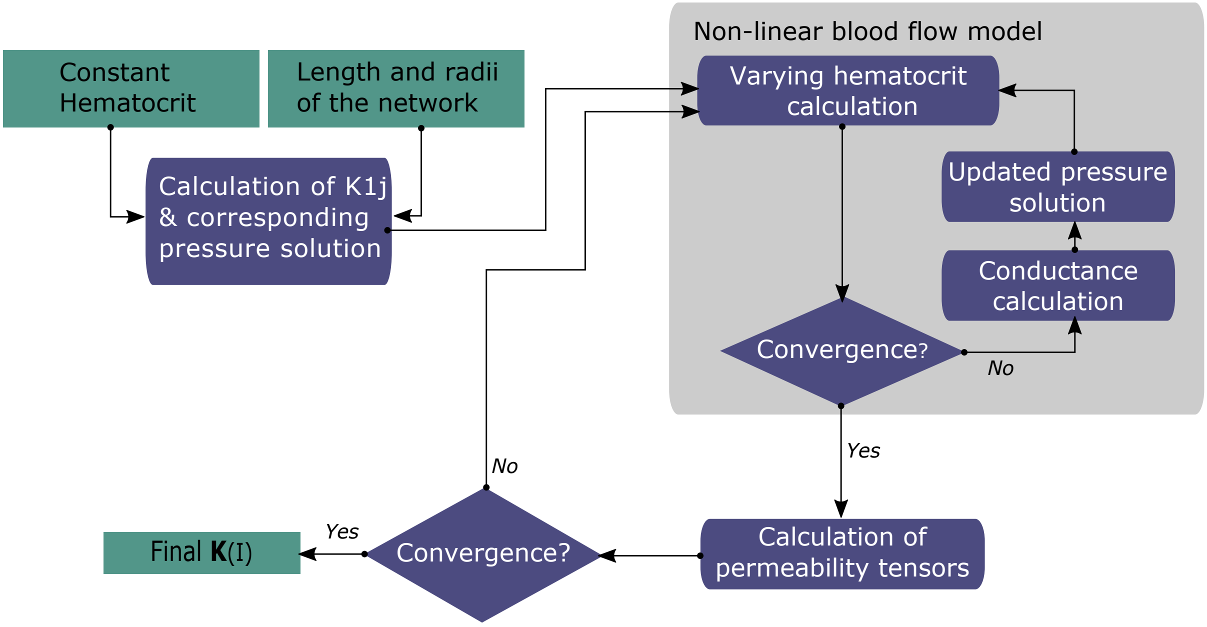

Figure S2. Overview of the approach for incorporating the phase sepa-ration effect in the calculation of permeability tensors.

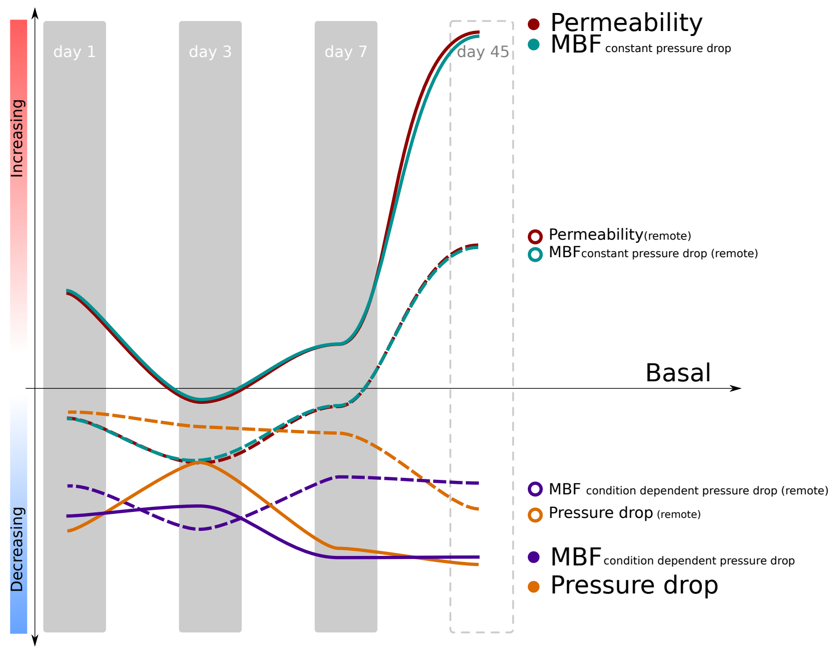

Figure S3. Summary of the changes in microvascular conductivity, AV pressure drop and MBF after MI. Dashed lines correspond to remote areas, while solid to infarcted. The scheme highlights the bimodal changes obtained for the per-meability tensors which increase at day 1 and day 7 after MI with partial restora-tion at day 3 (closer to basal). MBF calculated assuming physiological AV pressure drop independently of tissue condition, i.e. 19mmHg, follows the bimodal increase of the tensors at infarcted areas (blueish green line). On the contrary, when condition-dependent AV pressure drop is used, MBF remains reduced at infarcted areas (pur-ple line).

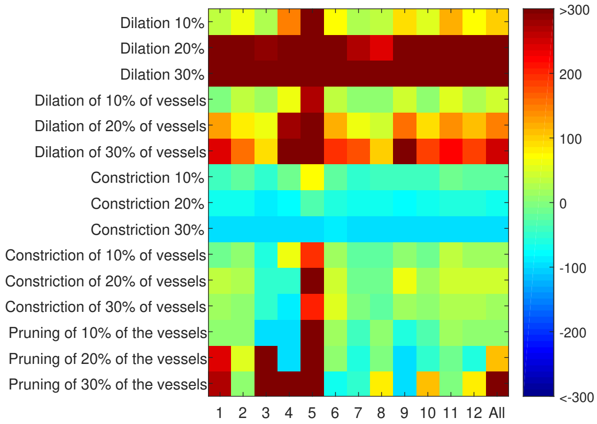

Figure S4. Impact of vascular remodeling strategies on the microvas-cular conductivity obtained by simulations using images of basal conditions. The pseudo-colored map represents the impact, i.e. percentage of change (%) of k_{11}, on the permeability tensors of the simulations for distinct vascular remodeling strategies described on the left. Every column corresponds to an image with the last column representing the mean among all images for the specific tissue condition.

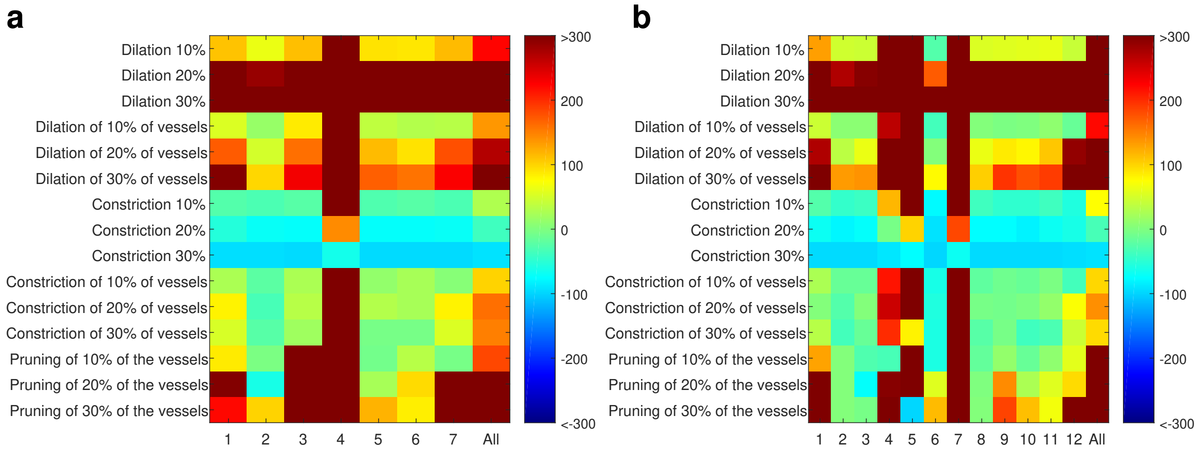

Figure S5. Impact of vascular remodeling strategies on the microvascu-lar conductivity obtained by simulations using images on day 3 post-MI. The pseudo-colored map represents the impact, i.e. percentage of change (%) of k_{11}, of the simulations for distinct vascular remodeling strategies described on the left. **a** Change in k_{11} when vascular remodeling is simulated using images from remote areas on day 3 post-MI. **b** Change in k_{11} when vascular remodeling is simulated for images from infarcted areas on day 3 post-MI. Every column corresponds to an image with the last column representing the mean among all images for the specific tissue condition.

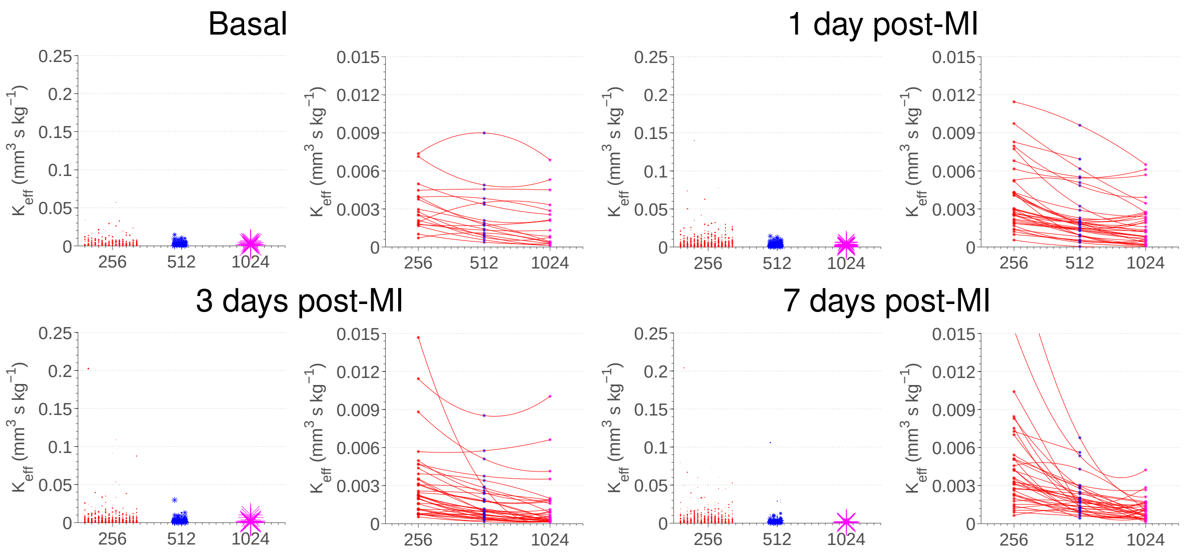

Figure S6. Dependency of the permeability tensor on Representative Volume Element. Three different sizes were investigated: $256 \times 256\times$ Nz, $512 \times 512\times$ Nz, $1024 \times 1024\times$ Nz voxels, with Nz standing for the size of the image along z-axis. The original image volume was therefore decomposed into 16, 4, and one volume(s)/unit(s) respectively depending on the unit size used. Tensors for the sub-volumes under investigation were calculated by applying the proposed approach for the calculation of permeability tensors from anatomical data on each sub-volume. The asterisk size is proportional to the volume of the units normalized by a volume size of $0.0074mm^3$ corresponding to $1024 \times 1024 \times 50$ voxels of size $[0.379, 0.379, 1007]\mu m$. The different asterisk sizes are due to the fact that the resulting connected network inside the unit might be smaller than the initial unit to which the image was decomposed. For each time point, the plots on the left show the maximum element of the permeability tensor in relation to volume size of the sub-networks of all images available in our dataset. On the right, there are plots for every image (one line per image) after fusing the tensors of the units in which it was decomposed. The permeability tensor here is given as the median of the different sub-volumes for simplicity reasons.

Supplemental References:

(1) El-Bouri WK and Payne SJ. Multi-scale homogenization of blood flow in 3-dimensional human cerebral microvascular networks. *Journal of Theoretical Biology*. 2015; 380:40–47.

(2) Auriault JL. Heterogeneous periodic and random media. are the equivalent macroscopic descriptions similar? *International Journal of Engineering Science*. 2011; 49:806 – 808.

(3) Shipley RJ and Chapman SJ. Multiscale modelling of fluid and drug transport in vascular tumours. *Bulletin of Mathematical Biology*. 2010; 72:1464–1491.

(4) Smith AF, Secomb TW, Pries AR, Smith NP, and Shipley RJ. Structure-based algorithms for microvessel classification. *Microcirculation*. 2015; 22:99–108.

(5) Secomb T. W. Simulation of microvascular network hemodynamics. `https://physiology.arizona.edu/people/secomb/netflow`.

(6) Pries AR and Secomb TW. Blood flow in microvascular networks. *Compr Physiol 2011, Supplement 9: Handbook of Physiology, The Cardiovascular System, Microcirculation*. 2008; 3–36.

(7) Pries AR, Secomb TW, Gessner T, Sperandio MB, Gross JF, and Gaehtgens P. Resistance to blood flow in microvessels in vivo. *Circ. Res*. 1994; 75:904–915.

(8) Baskurt O, Neu B, and Meiselman H.J. *Red Blood Cell Aggregation*. CRC Press, 2011.

(9) Gkontra P, Norton K-A, Żak MM, Clemente C, Agüero J, Ibáñez B, Santos A, Popel AS, and Arroyo AG. Deciphering microvascular changes after myocardial infarction through 3d fully automated image analysis. *Scientific Reports*. 2018; 8:1854.

(10) Creative commons license. `http://creativecommons.org/licenses/by/4.0/`.
